# Oriented Dipoles in Ordered Ensembles of Confined Lead Halide Perovskite Nanocrystals

**DOI:** 10.1021/acs.jpcc.5c07457

**Published:** 2026-01-26

**Authors:** Lindsey E. Parsons, Alexandra Y. Grishchenko, Carissa N. Eisler

**Affiliations:** Department of Chemical and Biomolecular Engineering, 8783 University of California, Los Angeles, Los Angeles, California 90095, United States

## Abstract

Here, we demonstrated directed self-assembly of quasi-2D cesium lead bromide perovskite nanoplates by liquid–air interfacial assembly. Due to their ionic crystal nature, perovskite nanocrystals are susceptible to degradation by most polar immiscible sublayer solvents (acetonitrile, diethylene glycol). We used glyceryl triacetate as a liquid substrate for nanoplate self-assembly. By tuning the interfacial energy and volume fraction of nanoplates, we achieved monolayer ensembles of nanoplates with face-down and edge-up ordering >100 μm^2^ in area. Controlled ordering of these confined structures allowed us to access aligned electronic transition vectors in perovskite nanocrystal thin films. The dipole orientation factor, or proportion of horizontal dipoles, was modulated from Θ = 0.78 for face-down assemblies to Θ = 0.48 for edge-up assemblies, which corresponds to the majority in-plane and out-of-plane emissive modes, respectively. Control over nanoparticle ordering and dipole orientation in perovskite nanocrystals could lead to enhanced waveguides, light outcoupling, and photon coherence in photonic devices.

## Graphical abstract

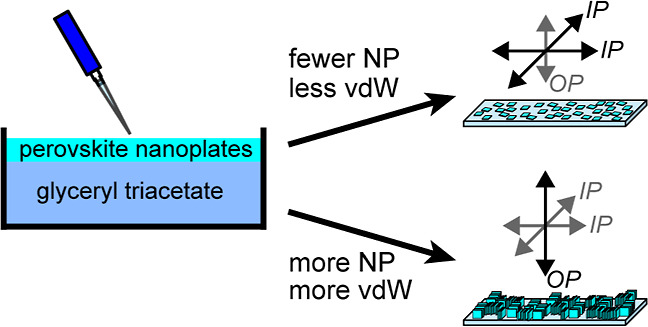

## Introduction

Liquid–air interfacial assembly is a common method to obtain highly ordered assemblies of nanoparticles across large size scales (100s of μm to cm).^1–7^ Interfacial and intermolecular forces can be tuned to access an energetic minimum to achieve the desired regime of assembly, such as assembling quantum dots (artificial atoms) into superlattices with specific crystal structures or ordering anisotropically shaped particles.^3,7–9^ This can be accomplished by tuning particle concentration^1,10^ or by adding unbound ligands into the sublayer that creates attractive or repulsive van der Waals (vdW) intermolecular forces at the interface.^2,4^ Additionally, nanoparticles can be “trapped” into a certain regime if their native solvent evaporates faster than the rate of assembly.^9^ For example, this method has been used to achieve face-to-face versus edge-to-edge assembled monolayers, which has unlocked advanced functionality in robust chalcogenide nanoplates like enhanced nonradiative energy transfer and tunable dipole orientation of the ensemble.^4,9^

Perovskite nanocrystals of varied morphologies have garnered broad attention as a next-generation semiconductor as they are a revolutionarily bright emitter with remarkable defect tolerance and fast exciton diffusion.^11–14^ There has been recent success in obtaining uniform, ordered films of the isotropic shapes of these perovskite nanoparticles. Notably, Baranov et al.^15^ demonstrated assembly of isotropically shaped perovskite nanocubes into 3D superlattices using perfluorodecalin as a sublayer, and Cherniukh et al.^8^ demonstrated assembly of perovskite nanocubes into monolayer films or into binary monolayer films along with Fe_3_O_4_ nanoparticles using glyceryl triacetate as a sublayer. However, for anisotropic morphologies in particular, their assembly at the liquid–air interface has had limited efficacy to date due to their ionic crystal nature and degradation in polar solvents (e.g., diethylene glycol, acetonitrile).^16–18^ For perovskite nanoplates, limited control of the edge-up versus face-down regime has been achieved with other methods. These include controlling the evaporation rate of the native solvent when the colloid is drop-cast onto a solid substrate,^19,20^ controlling the passivation of nanoplate surface charges,^21^ or by introducing horizontal shearing via spin-coating.^22^ Here, we demonstrated large-area (>100 μm^2^), conformal monolayers of oriented CsPbBr_3_ nanoplates through liquid–air interfacial assembly. We achieved control over the regime of assembly via the addition of oleic acid in the sublayer and by modifying the volume fraction of nanoplates used, resulting in face-down or edge-up orientations. Films of CsPbBr_3_ perovskite nanoplates were characterized by transmission electron microscopy (TEM) as well as UV–vis spectrophotometry and atomic force microscopy (AFM) to confirm the orientation and quantify film behavior. Angular emission of assembled thin films was characterized using Back-Focal-Plane Fluorescence Microscopy (BFPFM), which demonstrated the effect of controlled assembly on the effective dipole orientation of the film, which exhibits different alignment compared to other nanocrystal films (e.g., chalcogenides).

## Methods

### CsPbBr_3_ Nanoplate Synthesis

CsPbBr_3_ perovskite nanoplates were synthesized via a hot injection approach adapted from Bertolotti et al.^23^ In brief, cesium carbonate was heated with oleic acid under inert conditions to form cesium oleate. Lead bromide in mesitylene was dried under vacuum at room temperature and then solubilized using oleic acid and oleylamine while being heated under inert conditions. The temperature was changed to 99 °C, and cesium oleate was injected. The reaction was immediately quenched, the cyan colloidal product was cleaned, and the size was selected for further use. We note that a narrow size distribution obtained from careful size selection was necessary to observe ordered assemblies of the nanoparticles.

The nanoplates were 14 ± 3 nm wide laterally (*L*) and 4 ± 1 nm thick (*t*) as characterized by TEM, corresponding to a thickness of 6 monolayers.^23,24^ Figure 1a shows a TEM image of drop-cast nanoplates, which shows no large-scale organization (both edge-up and face-down are present). Figure 1b shows the absorbance of the nanoplates, which had two excitonic peaks at 431 and 470 nm, and a relative photoluminescence (PL) spectrum revealing a narrow emission centered at 490 nm with a full width at half-maximum (fwhm) of 18.8 nm. Typical synthetic variability resulted in emission centered at 488–492 nm.

**Figure 1:**
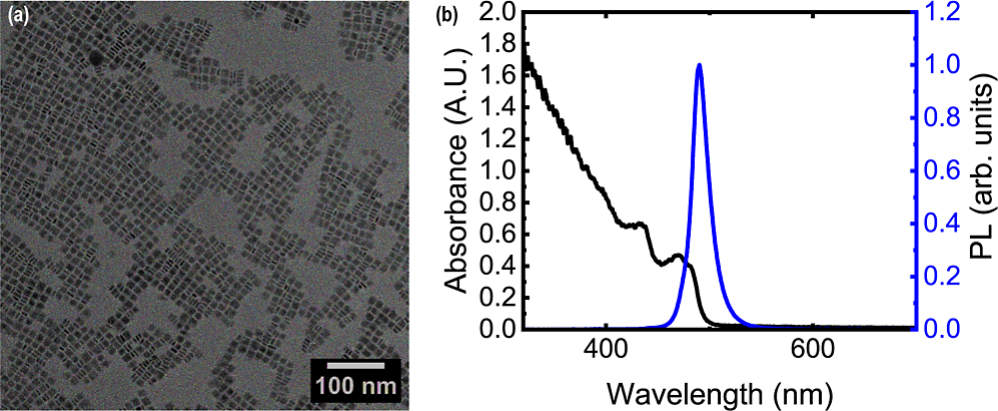
(a) Transmission electron micrograph showing as-synthesized CsPbBr_3_ nanoplates with an average length of 14 ± 3 nm and a thickness of 4 ± 1 nm. Plates can be seen in both the edge-up and face-down configurations when drop-cast. (b) Absorbance and relative photoluminescence of CsPbBr_3_ nanoplates emitting at 490 nm.

## Results and Discussion

### Nanocrystal Assembly at the Liquid–Air Interface

We used liquid–air interfacial assembly to control the orientation of anisotropic perovskite nanoplates in a monolayer assembly and, by extension, the effective dipole orientation of the film, as demonstrated in other material systems.^4,9^ In this approach, Brownian motion in the sublayer aids in achieving nanoparticle assembly into a thermodynamically favorable packing motif as the native solvent evaporates. The film is then transferred from the liquid sublayer to a solid substrate via controlled draining of the sublayer or via stamping the substrate onto the interface.^4,7^ Sublayer choice can be used to directly tune the interfacial energy between particles and the sublayer: Momper et al.^9^ used acetonitrile to achieve an edge-up assembly as opposed to diethylene glycol, which delivered a face-down assembly. Additionally, sublayer choice can lower the contact angle between the native solvent and the sublayer, where a greater wettability is more desirable.^8^

An additional consideration in selecting a sublayer for use with perovskite nanoplates specifically was their rapid degradation in most polar solvents. A screening of sublayer candidates measured the relative photoluminescence of CsPbBr_3_ nanoplates in hexane upon the small addition of each sublayer candidate. We tracked PL decay and red-shifting over time as indicators for sample degradation and fusing, respectively. Typical sublayers (acetonitrile, diethylene glycol) showed significant loss of emission and red-shifting, indicating degradation and fusing into larger particles. Fluorinert FC-40 showed the greatest stability (minimal PL reduction, no red-shifting) but was not selected based on the wettability considerations discussed in Cherniukh et al. for perfluorodecalin^8^ and due to the environmental and health concerns of fluorinated solvents.^25,26^ Glyceryl triacetate (GTA) was selected as the sublayer since it produced minimal red-shifting, and perovskite nanoplates in glyceryl triacetate maintained >70% of their initial emission intensity versus <10% in acetonitrile or diethylene glycol. See Supporting Information for further details.

For assembly, nanoplates in hexane were diluted to an optical density of 1.75 at 335 nm. GTA (containing a desired concentration of oleic acid) was added to a 60 mm diameter glass Petri dish, followed by a given volume (usually 100–200 μL) of the CsPbBr_3_ nanoplate suspension, which was tuned to control the final volume fraction of nanoplates in the assembly. The dish was immediately covered to standardize the rate of solvent evaporation and protect the assembly process from outside movement of the surrounding gas. Once the native solvent was completely evaporated (less than 5 min), the cover was removed, and the assembled film was transferred to the desired substrate (TEM grid or untreated 22 × 22 mm borosilicate glass coverslip). A schematic of the assembly is given in Figure 2a. See Supporting Information for further details. Note that films were fabricated inside an argon glovebox to avoid detrimental oxygen and water exposure during assembly. Additionally, the glovebox purifiers were momentarily turned off, and the glovebox was not allowed to refill to minimize airflow and reduce film disturbance.

**Figure 2:**
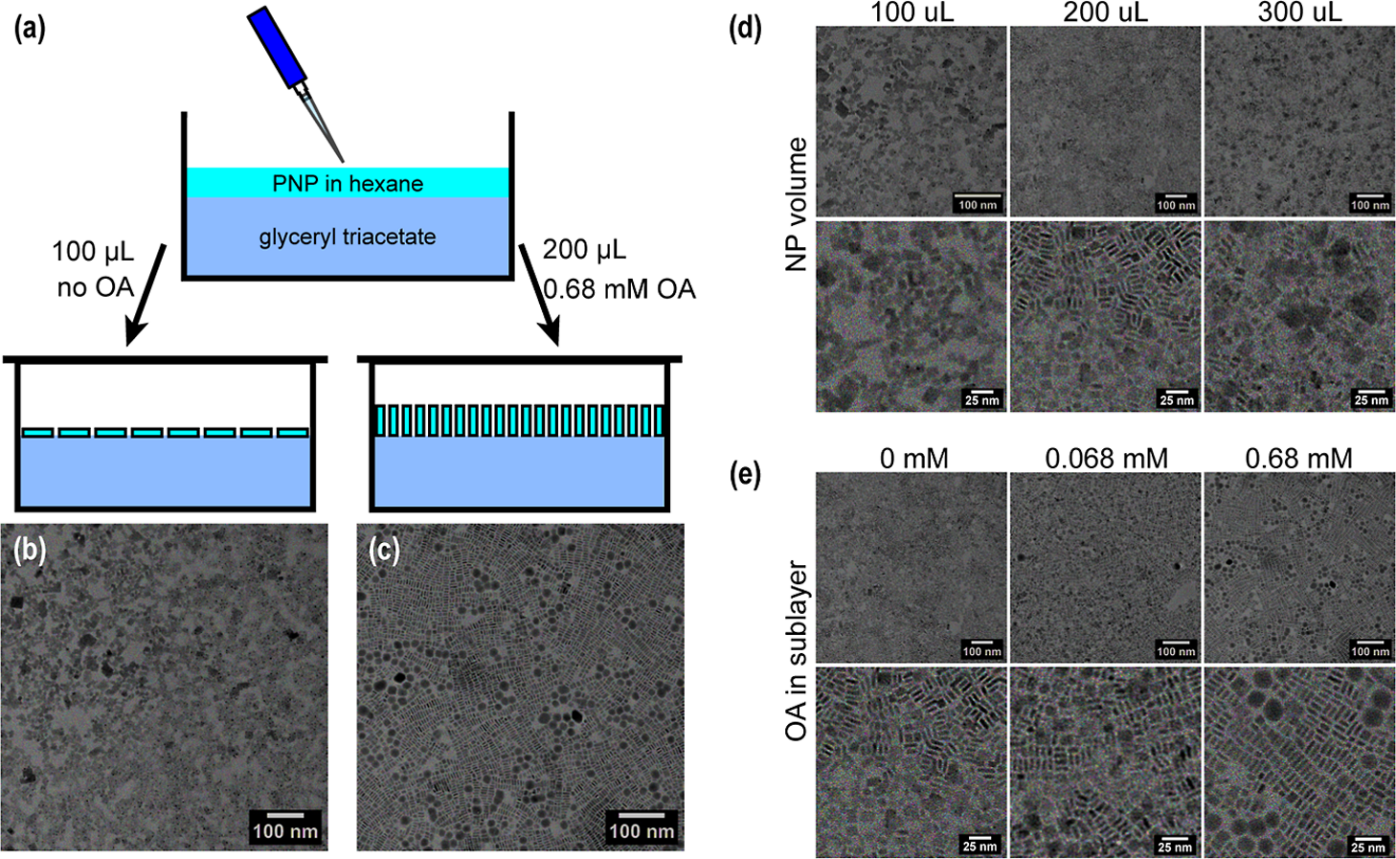
(a) Schematic of assembly at the liquid–air interface on a glyceryl triacetate (GTA) sublayer. GTA forms an immiscible sublayer with hexane, providing a liquid substrate on which nanoplates self-assemble upon evaporation of hexane. Transmission electron micrographs showing CsPbBr_3_ nanoplates assembled in a (b) face-down and (c) edge-up configuration. TEM showing the regime of assembly for (d) increasing nanoplate volume fraction (100–300 μL added) with no OA and (e) increasing OA concentration in the sublayer (0 mM–0.68 mM) for a 200 μL nanoplate volume.

Here we explored how the addition of ligands to the sublayer and the total volume of particle solution added affected the order and assembly of the anisotropic perovskite nanoplates. Nanoplates oriented face down have a larger surface area footprint than nanoplates oriented edge up (around 190 nm^2^ versus 52 nm^2^, respectively, exclusive of interparticle spacing and ligand chain length). Thus, we hypothesized that the volume fraction of nanoplates in the assembly could be tuned to achieve the desired phase, where a higher volume (more total particles) would enable more edge-up packing, while a smaller volume (fewer total particles) would enable more face-down packing. Such concentration-induced ordering is a well-understood phenomenon borrowed from the field of lyotropic liquid crystals and adapted for our purposes.^10^ By changing the volume of nanoplate suspension added, we effectively changed the volume fraction of the nanoplates in the assembly. We observed that increasing the volume fraction of nanoplates increased edge-up packing (Figure 2d). At lower volume fractions (100 μL), face-down orientation was achieved most readily. Increasing the volume fraction resulted in a mix of orientations due to the energetic minimization of close packing, and at very high volume fractions (300 μL), aggregation and vertical stacking of plates were observed (Figure S6).

We hypothesized that a small addition of oleic acid (OA) into the GTA sublayer could be used to also tune the orientation of the plates in the assembly by increasing the repulsive van der Waals (vdW) forces between the oleic acid/oleylamine-functionalized nanoplates and the sublayer.^27^ This was reported in other nanocrystal systems to both tune the orientation and prevent nanoparticle aggregation.^4^ We observed a transition to edge-up assembly when the OA concentration was increased for a consistent volume of nanoplates (200 μL) (Figure 2e). We note that the optimum concentration was 0.68 mM OA as nanoplates assembled into edge-up, nematic assemblies. At lower concentrations of OA (i.e., 0.068 mM), we observed a mix of edge-up and face-down particles, and at concentrations beyond 0.68 mM, we observed macroscopic aggregation on the surface of GTA. We observed that adding both oleic acid and oleylamine to the sublayer yielded more undesirable aggregation than oleic acid alone (Figure S8).

In summary, we tuned the knobs of ligand concentration and nanoplate volume fraction to demonstrate control between the face-down and edge-up regimes. A smaller volume fraction in the resulting assembly (100 vs 200 μL) thermodynamically favored a face-down assembly due to unconstrained particle packing. The addition of OA to the sublayer (0.68 mM vs 0 mM) introduced repulsive vdW forces, which induced an edge-up configuration and reduced aggregation. Figure 2b,c shows face-down and edge-up ensembles that will be further characterized in the following sections.

### Thin Film Characterization Confirming Ordering

For further characterization, we used a nanoplate colloidal volume of 100 μL and no OA in the sublayer to obtain a face-down assembly and a nanoplate colloidal volume of 200 μL and 0.68 mM OA in the sublayer to obtain an edge-up assembly. After transferring the assembled films to glass coverslips, face-down and edge-up ensembles of CsPbBr_3_ nanoplates were characterized to corroborate the regime of ordering and characterize them both optically and structurally. Relative photoluminescence of face-down and edge-up films showed emission centered at 493–496 and 491–496 nm, respectively (Figure 3a). TEM was used to estimate the packing fraction of the face-down and edge-up regime at the nanoscale using a Bruggeman effective medium approximation for nanocrystal versus ligand area,^28,29^ which resulted in an effective refractive index of 1.78 for face-down assemblies and 1.87 for edge-up assemblies. Figure 3b,f shows the TEM image of larger assembled areas of the face-down and edge-up regime, generally showing large-scale assemblies. Figure 3f also highlights nanoscale aggregations at the edge of these assemblies that inevitably result in slight variability of thickness in the edge-up regime.

**Figure 3:**
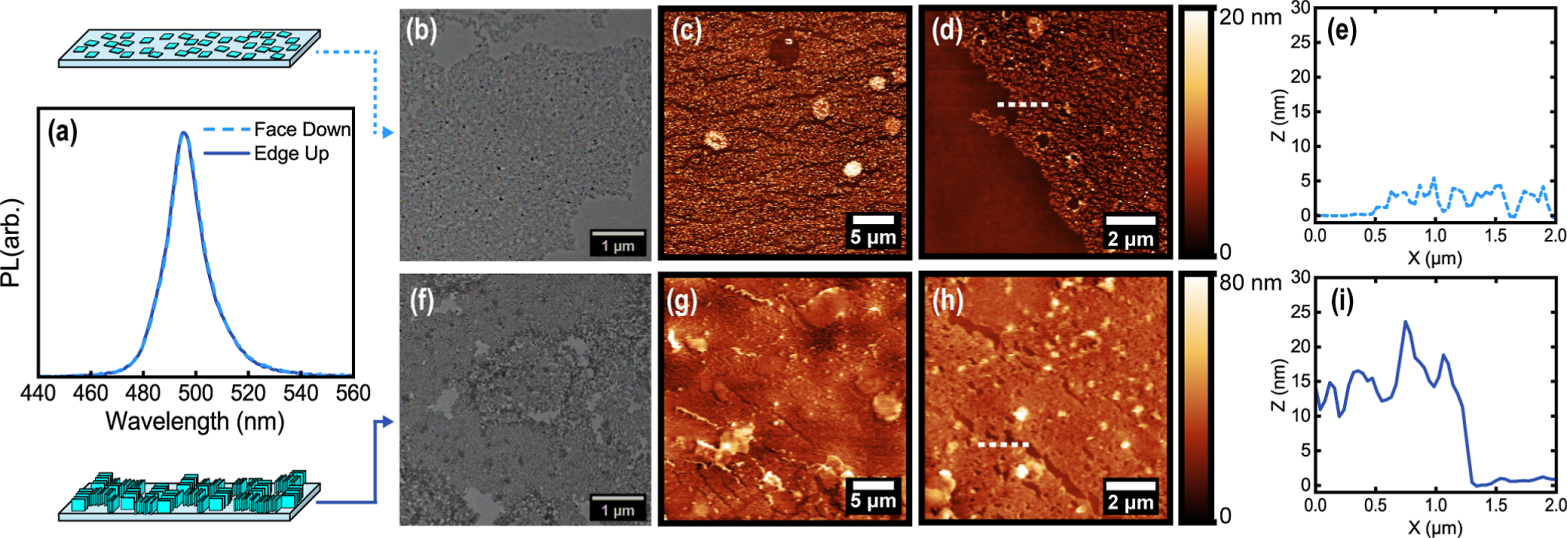
(a) Relative photoluminescence of face-down and edge-up assembled films showing emission at 496 and 495 nm, respectively. For face-down assemblies, (b) large-area TEM showing interparticle spacing and (c–e) atomic force micrographs and extracted cross section showing film uniformity on a micron scale. The white line denotes where the 2 μm cross section was extracted. The average height of face-down assemblies was 3.4 nm from AFM. For edge-up assemblies, (f) large-area TEM showing interparticle spacing and (g–i) atomic force micrographs and extracted cross section showing film uniformity on a micron scale. The white line denotes where the 2 μm cross section was extracted. The average height of edge-up assemblies was 15.2 nm from AFM.

AFM was used to characterize film uniformity and thickness at the micron scale. Prior work on aligning anisotropic perovskite nanoparticles through drop-casting and spin-coating has generally demonstrated good alignment and ordering; however, these methods have been limited to either smaller domain sizes (<100s nm)^19,20,22^ or to thicker films with multilayer stacks of particles.^21,22^ Here, we demonstrate much larger domain sizes (>100 μm^2^) of densely packed nanoparticles in a single monolayer film. Figure 3c–e shows the AFM scans and extracted profile for the facedown assembly, and Figure 3g–i shows the AFM scans and extracted profile for the edge-up assembly. The thicknesses of these large-area assemblies corroborated with the expected thicknesses for face-down and edge-up nanoplate monolayer assemblies. From AFM, face-down ensembles of nanoplates had an average thickness of 3.4 nm, and edge-up ensembles had an average thickness of 15.2 nm. This was in excellent agreement with the TEM particle sizing of a 4 ± 1 nm thickness and a 14 ± 3 nm edge length. Locally, uniform domains of oriented particles of >100 μm^2^ in area were observed, although some film tears and aggregates were present, which prevented realizing much larger domains (cm^2^) of oriented particles. Both TEM and AFM revealed more gaps in the face-down films at the nanometer and micrometer scale as compared to the edge-up films, which was expected due to the lesser total volume fraction of nanoparticles. Thicker areas and aggregations were observed in edge-up assemblies after being transferred to a solid substrate, particularly the untreated glass coverslips (Figure S11). Additionally, a coffee-ringing effect was observed at the micrometer scale in samples where excess GTA sat before being evaporated under vacuum (Figure S12). In the subsequent optical study of edge-up assemblies, regions that clearly had large aggregates or defects were not included in the characterization (see Supporting Information). Surface treatments to increase the hydrophobicity of the substrate could potentially combat this substrate-induced aggregation and film disruption, yielding larger areas of oriented films than the >100 μm^2^ oriented area films seen here.

### Resultant Oriented Dipoles

We characterized the alignment of dipoles using Back-Focal-Plane Fluorescence Microscopy (BFPFM), which measures the intensity of photoluminescence as a function of angle or momentum. This is accomplished by inserting a Bertrand lens into an inverted fluorescence microscope, which transforms the focal plane of the signal, according to the procedure described by Kurvits et al.^30^ After changing the focus to the back-focal plane, the angular emission signal is fit to a three-layer model of an emissive film as a function of momentum vectors, where the emission intensity (*N*_pol_) in *k*-space (*k*_*x*_, *k*_*y*_) is given as^31,32^

1 $$N_{pol}\left(k_{x},k_{y}\right)=C\left[\frac{1}{2}\left(\rho_{ip}^{p}+\rho_{ip}^{s}\right)\cos^{2}\left(TDM\right)+\rho_{op}^{p}{\;\sin}^{2}\left(TDM\right)\right]$$

where *C* is a normalization constant; ρ_ip_^s^, ρ_ip_^p^, and ρ_op_^p^ are the photonic density of states in the s- and p-polarized in-plane and out-of-plane directions governed by the thickness (*D*), refractive index (*n*_film_), and emission wavelength of the emissive layer; and TDM is the effective angle of electronic transitions in the emitting layer, measured from the plane of the substrate in the *z* direction. TDM can be related to the dipole orientation factor Θ, which is the ratio of in-plane dipole contributions (*p*_∥_) to all dipole contributions (*p*_∥_ + *p*_⊥_).^33,34^

2 $$TDM=\arccos\left(\sqrt{\Theta }\right)$$

3 $$\Theta =\frac{p_{∥}}{p_{∥}+p_{⊥}}$$

The angular emission patterns are a strong function of dipole orientations and thin film properties (thickness and refractive index). Figure 4a,c shows how the signal evolves as a function of TDM for a film with thickness = 0 and 15 nm, respectively. As TDM increases, the intensity at |*k*_*x*_| ≥ 1 increases; this is most clearly illustrated in the p-polarized cross section of the signal (*k*_*y*_ = 0) (Figure 4b). The s-polarized cross section of the signal (*k*_*x*_ = 0) changes only in response to changes in the photonic density of states (thickness, RI, λ). Figure 4d shows the s-polarized cross section for varying thickness, showing how the location of maximum intensity in *k*-space (|*k*_*y*,max intensity_|) increases with increasing thickness of the emitting layer.^29^ Details of BFPFM and dipole orientation fitting are provided in the Supporting Information.

**Figure 4:**
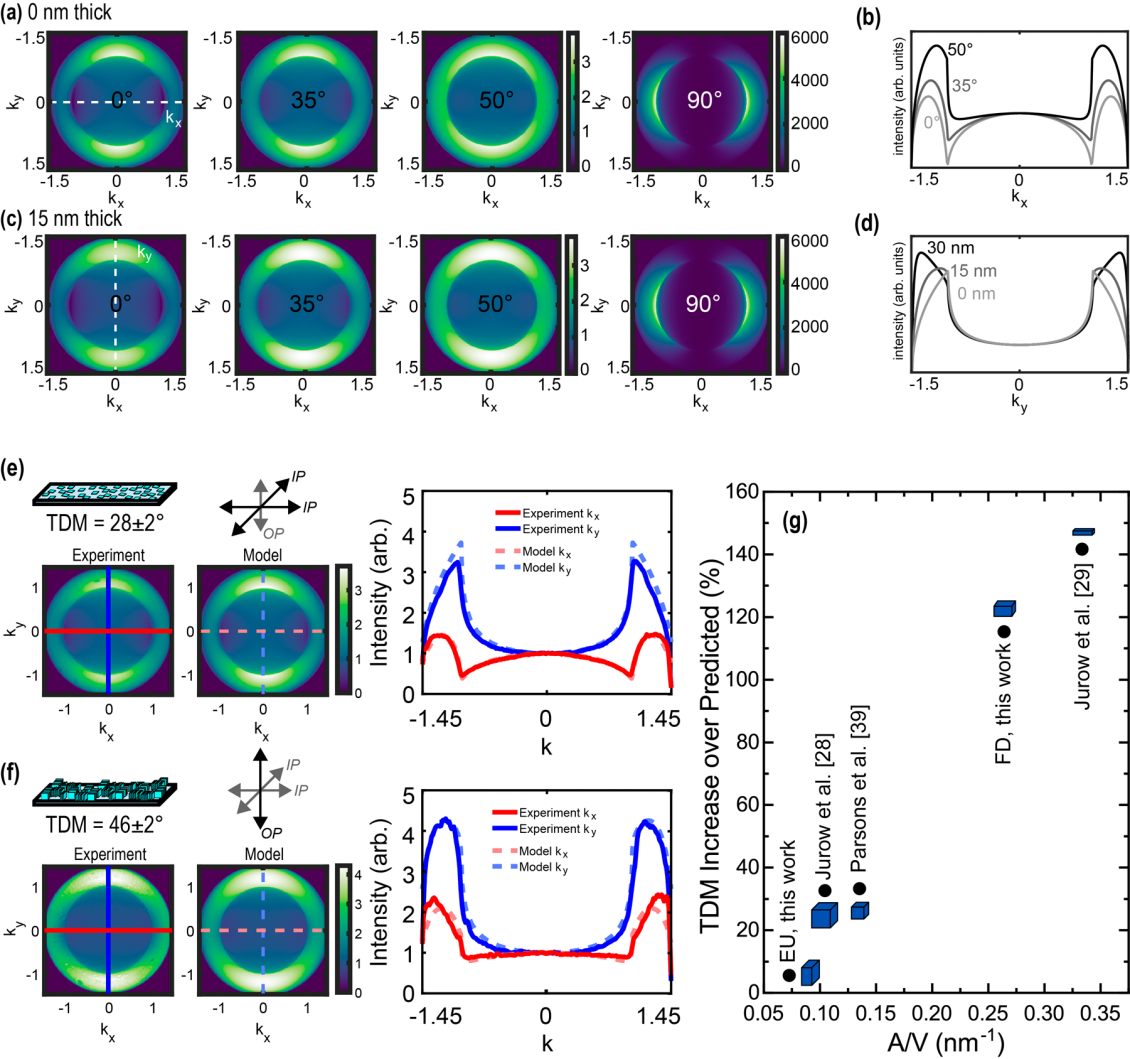
Modeled BFPFM signal for an emissive layer (a) 0 nm thick and (c) 15 nm thick with an effective transition dipole moment of the ensemble at 0° (in-plane contributions only), 35° (isotropic), 50°, and 90°(out-of-plane contributions only). (b) *k*_*x*_ cross section, where *k*_*y*_ = 0 for varying TDM. (d) *k*_*y*_ cross section, where *k*_*x*_ = 0 for varying thicknesses. (e) Collected BFPFM signal, modeled signal of best fit, and extracted *k*_*x*_ (red) and *k*_*y*_ (blue) cross sections for face-down assemblies with an effective TDM of the ensemble at 28 ± 2° or Θ = 0.78, corresponding to stronger in-plane dipole contributions. (f) Collected BFPFM signal, modeled signal of best fit, and extracted *k*_*x*_ (red) and *k*_*y*_ (blue) cross sections for edge-up assemblies with an effective TDM of the ensemble at 46 ± 2° or Θ = 0.48, corresponding to stronger out-of-plane dipole contributions. (g) Increase in TDM of a monolayer of CsPbBr_3_ nanocubes and nanoplates on glass compared to theoretical predictions for this work and prior work, plotted against particle contact area with the substrate scaled by particle volume (nm^–1^).^28,29,39^ Data reproduced from ref 28. Copyright 2017 American Chemical Society. Data reproduced from ref 29. Copyright 2019 American Chemical Society. Data reproduced from ref 39. Copyright 2025 American Chemical Society.

For an ensemble of completely confined, ideal 2D plates oriented in-plane, the expected TDM would be 0° or Θ = 1, eliminating out-of-plane dipole contributions. Ideal 2D plates oriented out of plane would have an expected TDM of 45° or Θ = 0.5. Previous work accomplished tunability over this entire range (Θ = 1 to Θ = 0.5) using strongly confined 2D CdSe nanoplates.^4^ Generally, the TDM for face-down perovskite nanoplates has been shown to be a function not only of confinement but also of composition, ligand choice, electronic environment, and film thickness.^35–38^ For CsPbBr_3_ perovskite nanoplates (4 nm thick, aspect ratio *t*/*L* = 0.28), prior modeling work by Jurow et al.^29^ and Marcato et al.^22^ predicted a TDM of 13° (Θ = 0.95) and 15.5° (Θ = 0.93), respectively, for face-down orientations. For edge-up orientations, Marcato et al.^22^ predicted a TDM of 43.5° (Θ = 0.53), and predicted TDM values reported by Jurow et al.^29^ can be translated to a TDM of 43.5°(Θ = 0.53) for edge-up orientations. The reduced tunability is due to the fact that, unlike the CdSe nanoplates, these nanocrystals are not fully confined, retaining a smaller dipole contribution along the thinnest dimension. Prior modeling work shows that horizontal dipole orientation (TDM approaching 0°, Θ approaching 1) in isolated perovskite nanoplates would only be achieved in extremely thin, high-aspect-ratio nanoplates (particle thickness <2 nm, particle length >20 nm).^22^ For lightly confined perovskite nanoplates around 3 nm thick, Jurow et al. reported a measured TDM of 29° (Θ = 0.78) for a monolayer film of face-down plates on glass.^29^ This angle was further increased beyond what was predicted (TDM = 13°) because of a characteristic enhanced vertical dipole contribution in response to charge at an interface for perovskite nanocrystals.^28,29,39–41^ We anticipated a larger vertical dipole contribution for edge-up plates over face-down plates due to the orientation of the thinnest dimension, and we anticipated an enhancement of the vertical dipole contribution in both regimes relative to prior modeling efforts due to the glass interface.

Figure 4e,f shows the collected BFPFM signal and modeled signal of best fit for the face-down and edge-up assembly regimes. |*k*_*y*,max intensity_| for *k*_*x*_ = 0 was greater for edge-up assemblies than face-down assemblies, corroborating other comparative measurements of thickness between regimes. Face-down assemblies were fit using a model thickness of 0 nm (phase shift is neglected), which is an accepted approximation when the thickness is much smaller than the wavelength of light (film thickness from AFM = 3.4 nm),^4,28^ and an RI of 1.78 from effective medium approximation. Edge-up assemblies were fit using a model thickness of 15 nm in accordance with the average film thickness from AFM (15.2 nm) and an RI of 1.87. Edge-up assemblies had more variability in BFPFM images due to the lateral size distribution of the nanoplates (14 ± 3 nm) and increased aggregation. As such, data that could not be reasonably fit to a 15 nm thickness were excluded from the reported average TDM fit for edge-up assemblies. Figure S16 shows the *k*_*y*_ cross sections of data that were excluded from the reported fit, as well as the average *k*_*y*_ cross section and modeled *k*_*y*_ cross section at 15 nm.

Signal intensity at |*k*_*x*_| ≥ 1 for *k*_*y*_ = 0 was smaller for face-down than for edge-up assemblies, implying a more horizontal dipole component for face-down assemblies. Ensembles of face-down CsPbBr_3_ nanoplates displayed a TDM of best fit of 28 ± 2° (Θ = 0.78), which corroborated previous reports of a monolayer of face-down nanoplates of similar nanoplate thickness on glass (29°, around 3 nm thick).^29^ The observed effective TDM angle (28 ± 2°) for the face-down assembly was greater than that for the ideal 2D plate oriented in plane (0°) because both particle geometry and surface effects served to enhance the vertical TDM contribution beyond ideal values, as discussed earlier. These plates were lightly confined, and thus, as expected, transitions along the out-of-plane thickness dimension of the plate were not completely eliminated. Additionally, their unique vertical dipole enhancement at a glass interface served to increase the TDM above even what a lightly confined geometry would suggest.^28,29,39–41^ Ensembles of edge-up CsPbBr_3_ nanoplates displayed a TDM of best fit of 46 ± 2° (or Θ = 0.48). The observed TDM was again higher than the expected 43.5°, which we attributed to the effect of interfacial charge generation on the ionic lattice. In order to increase the range of accessible dipole orientations (TDM approaching 0°, Θ approaching 1), two conditions would need to be met. First, moving to ultrathin (<2 nm), high-aspect-ratio nanoplates would minimize the dipole contribution along the thin axis.^22^ Second, the interfacial charge would need to be strongly reduced or eliminated to prevent a vertical dipole enhancement, which can be achieved either by selecting a substrate with a minimal work function difference to the perovskite nanoplates^39^ or by encapsulating the nanoplates in a similar material to the substrate and canceling out the substrate effect.^29^ We also observed that the surface effects had a much larger effect on the face-down assemblies compared to the edge-up assemblies. The observed TDM of 28 ± 2° for face-down assemblies was 81–115% greater than the TDM predicted by Marcato et al. and Jurow et al., while the observed TDM of 46 ± 2° for the edge-up orientations was only 6% greater.^22,29^ The contact area of the edge-up nanoplate is smaller than the contact area of the face-down nanoplate relative to the total particle volume, suggesting that the apparent effect of interfacial charge generation is diminished for edge-up nanoplates as opposed to face-down nanoplates based on diminished contact area. To illustrate this, we report the TDM increase (%) of CsPbBr_3_ nanocrystals on glass compared to predicted values as a function of the area-to-volume ratio (or the inverse of the sample thickness) for the films presented here as well as unconfined nanocube particles and face-down nanoplates from other studies (Figure 4g).^28,29,39^ We only compared this to other studies that characterized a single monolayer of the same material (CsPbBr_3_) to isolate the effect of surface interaction on orientation and area-to-volume ratio. The largest increases are observed for the thinnest films (face-up assemblies). This is intuitive because the enhanced vertical dipole contribution is an interfacial response, and thicker samples would feel the interfacial effects less strongly. This is also why increased nanoparticle spacing leads to an enhanced vertical dipole contribution as shown by prior work on varying surface coverages of monolayer perovskite nanocrystal films.^28^ According to this relationship, moving to smaller CsPbBr_3_ nanoparticles would be best suited to take advantage of the surface-induced dipole tunability. Further, increasing the separation of these particles, which can be done by using longer ligands, can potentially further exaggerate the surface effects.^42^

## Conclusion

We showed that by careful sublayer selection, assembly at the liquid–air interface can be applied to delicate inorganic lead halide perovskite nanocrystals. By tuning the knobs of volume fraction and interfacial energy via addition of an unbound ligand in the sublayer, we could direct monolayer assembly. We used a nanoplate colloidal volume of 100 μL and no unbound oleic acid in the sublayer to obtain a face-down assembly and a nanoplate colloidal volume of 200 μL and 0.68 mM unbound oleic acid in the sublayer to obtain an edge-up assembly. We then characterized momentum-resolved photoluminescence of the resultant films, uncovering the interplay of orientation and confinement—as well as characteristic response to surface charges—on the effective orientation of dipoles in 2D ensembles of CsPbBr_3_ nanoplates. Face-down assemblies of lightly confined nanoplates showed a TDM of 28 ± 2° (Θ = 0.78), while edge-up assemblies showed a TDM of 46 ± 2° (Θ = 0.48). Since the out-of-plane dipole contribution is not completely eliminated for a lightly confined plate, we did not expect to achieve dipole orientation control over the entire range of effective TDMs that are theoretically possible for 2D morphologies (0° to 45°).^4^ However, paired with the characteristically enhanced vertical dipole contribution of CsPbBr_3_ nanocrystals on glass, we can achieve a vertical effective TDM of 45°.

This method for achieving thermodynamically controlled nanocrystal assembly, while common in other material systems,^2,4,9^ has had limited success for perovskite nanocrystals.^8,15^ This work provides a promising route by which the technique may be leveraged for applications that benefit from both in-plane and out-of-plane specific modes of emission, such as solar concentrating technologies, LEDs, or quantum information technologies.^37,43–47^ With improved substrate compatibility, these assemblies could be realized over larger areas (>1 cm^2^), and for more extreme shapes approaching 1D (nanorods, nanowires), vertical assembly could enable nearly entirely in-plane light emission.

## Supplementary Material



## Notes

Detailed methods, additional structural and optical characterizations, and derivation of the PDOS model of angular light emission (PDF)
