# Supplementary material for: Oriented Dipoles in Ordered Ensembles of Confined Lead Halide Perovskite Nanocrystals
Source: J Phys Chem C Nanomater Interfaces. 2026 Jan 26;130(5):1996–2003. doi: 10.1021/acs.jpcc.5c07457 (PMC12884527; doi:10.1021/acs.jpcc.5c07457)
Supplement: Supplementary file 1 [file jp5c07457_si_001.pdf]

Supplemental Information for Oriented Dipoles in Ordered Ensembles of Confined Lead Halide Perovskite Nanocrystals

*Lindsey E. Parsons, Alexandra Y. Grishchenko, Carissa N. Eisler**

Department of Chemical and Biomolecular Engineering, University of California, Los Angeles, Los Angeles, California 90095, United States

*Carissa N. Eisler ceisler@ucla.edu

Materials 1
Nanoplate synthesis 1
Nanocube synthesis 2
Transmission Electron Microscopy 3
UV-vis spectrophotometry 4
Sublayer screening 4
Thin film preparation via assembly at the liquid-air interface 5
Atomic Force Microscopy 11
Assembly extended to $CsPbBr_3$ nanocubes with DDAB ligand 13
Back Focal Plane (BFP) Fluorescence Microscopy 14
Modeling angle-resolved light emission 15
Poor fits of BFPFM data indicating thickness variability and defects in assemblies 17
Effect of surface charging on nanoplate TDM 18

Materials

Lead (II) bromide ($PbBr_2$, 99.999%, Sigma Aldrich), cesium carbonate (Cs_2CO_3, 99%, Sigma Aldrich), oleic acid (OA, technical grade 90%, Sigma Aldrich), oleylamine (OAm, technical grade 70%, Sigma Aldrich), mesitylene (99%, Sigma Aldrich), hexane (anhydrous, ≥99%, Sigma Aldrich), ethyl acetate (anhydrous 99.8%, Sigma Aldrich), octanoic acid (OctAc, ≥98%%, Sigma Aldrich), 1-octyl phosphonic acid (OPA, 99%, Thermo Scientific), trioctylphosphine oxide (TOPO, 99% Thermo Scientific), didecyldimethyl ammonium bromide (DDAB, >98%, TCI), methyl acetate (MeAc, 99.5%, Sigma Aldrich), toluene (99.8%, Sigma Aldrich), heptane (99%, Sigma Aldrich), and glyceryl triacetate (≥99.0%, Sigma Aldrich) were used without further purification.

Nanoplate synthesis

Procedure was adapted from Bertolotti et al. [1] and Bekenstein et al.[2]

Cs-oleate precursor: 0.4 g Cs_2CO_3 was added to a round bottom flask along with 1.2 mL OA and 15 mL ODE. Flask was connected to the Schlenk line and degassed under vacuum for 1 hr at 100 °C, then switched to Ar and heated at 120 °C until all Cs_2CO_3 was dissolved and solution was brown in color, indicating that it had all reacted with OA.

Synthesis: 0.069 g $PbBr_2$ added to a 25 mL round bottom flask along with 5 mL mesitylene. Flask was connected to the Schlenk line and degassed at room temperature. Flask was then switched to Ar and heated to 100 °C, followed by the injection of 0.5 mL OA and 0.5 mL OAm. Once all $PbBr_2$ was dissolved, the temperature was changed to 99 °C, and 0.4 mL heated (to at least 100°C) Cs-oleate was injected into the PbBr2 precursor, and the reaction was immediately quenched with liquid nitrogen.

For a 2x scaled reaction, 0.138 g $PbBr_2$ was used, along with 1 mL OA and 1 mL OAm, 0.8 mL heated Cs-oleate. The injection was done at 125 °C.

Cleaning: Crude solution was divided into 3 15 mL tubes and centrifuged for 10 minutes at 11,000 rpm (14,881 g). Pellet was retained, and any supernatant that could not be sufficiently decanted by pipetting was gently removed using a cotton-tipped applicator. Pellet was redispersed in 100 µL hexane per tube and centrifuged again for 20 minutes at 11,000 rpm.

100 µL supernatant was added to two new tubes, along with 200 µL hexane and 600 µL ethyl acetate per tube. The tubes were centrifuged for 5 minutes at 11,000 rpm. Pellet was retained, and again any supernatant that could not be sufficiently decanted was removed using a cotton-tipped applicator. Pellet was redispersed in 100 µL hexane per tube and centrifuged again for 10 minutes at 11,000 rpm. The supernatant was filtered by a 0.22 µm nylon syringe filter and retained as product.

Nanocube synthesis

Procedure was adapted from Brown et al.[3]

Cs-octanoate precursor: 326 mg Cs_2CO_3 was added to 10 mL OctAc and vortexed until dissolved.

Pb precursor: 500 mg $PbBr_2$ was added to 10 mL toluene along with 5.8 g TOPO and heated to 80 °C while stirring until completely dissolved (around 15 minutes). Solution was allowed to cool completely to room temperature before use.

DDAB precursor: 233 mg DDAB was added to 10 mL toluene and vortexed until dissolved.

Synthesis: Under constant stirring, 2 mL of Pb precursor was added to round bottom flask. 233 mg of OPA was added and stirred at room temperature until dissolved to create a ligand concentration of 0.6 mM. In air and at room temperature, 220 µL Cs-octanoate precursor was injected, and the reaction was allowed to proceed for 30 seconds. 624 µL DDAB precursor was added, and the product was allowed to continue stirring for 5 minutes.

Cleaning: First 6 mL MeAc was added to the crude solution to clean the particles. The product was divided between 3 15 mLcentrifuge tubes and centrifuged for 20 minutes at 10,000 rpm (12298 g). The precipitate was retained and redispersed in 150 µL heptane per tube to then perform a knockout step. The three tubes were centrifuged for 1 minute at 5000 rpm (3354 g), and the supernatant was retained as product.

Transmission Electron Microscopy

Images were collected on a FEI Tecnai T12 microscope using 200 μm or 400 μm carbon coated copper grids.

TEM images in figure S1 were used to estimate nanoparticle packing in order to determine the effective film RI via Bruggeman Effective Medium Approximation as was done by Jurow et al. [4] For face down cases, after smoothing, despeckling, and increasing the contrast using ImageJ, nanoplate was estimated at 39% of the area, the average of three selected representative regions of best resolution. For edge up cases, nanoplate was estimated at 49% of the area. Perovskite nanocrystal has an RI of 2.3.[5] Space between particles comprising the remainder of the area was presumed to be ligand with an RI of 1.45 [6].

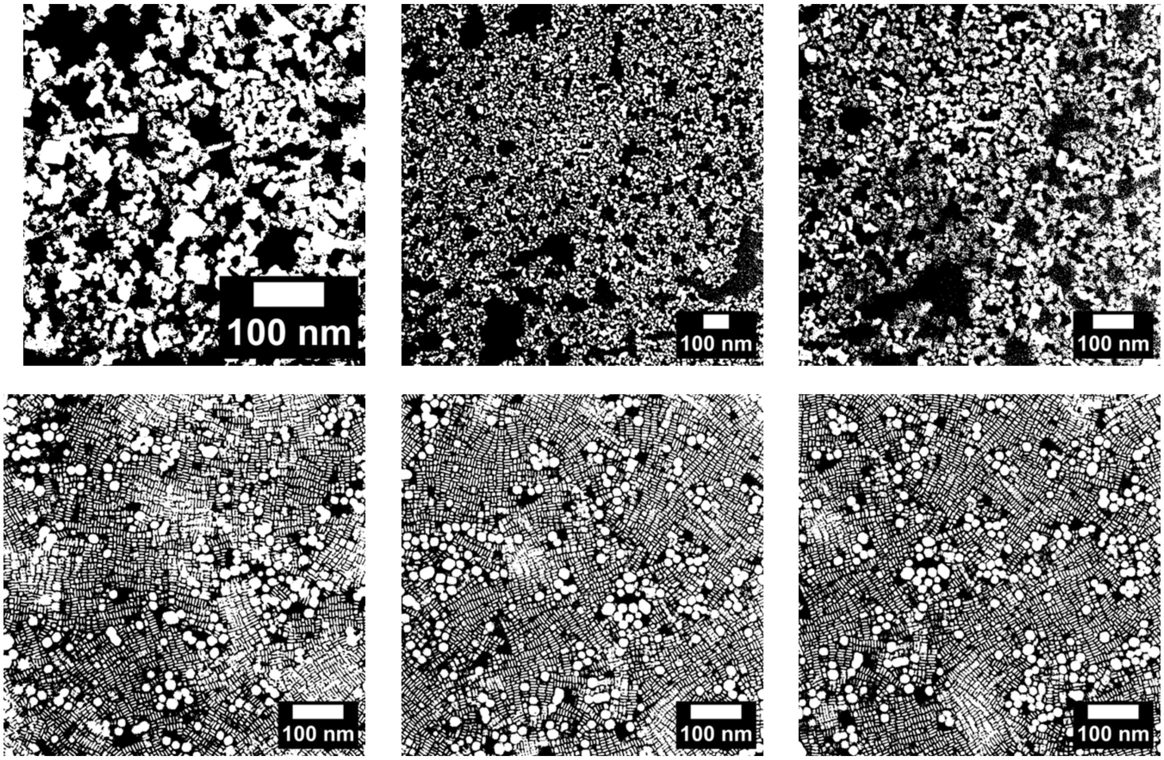

Figure S1. Contrast-enhanced TEM images used for effective medium approximation. Top row is face down samples; bottom row is edge up samples. White pixels are counted as nanocrystal area and black pixels are counted as ligand area.

The effective RI of the film was thereby estimated by the following equation:

$$RI = Packing\ Fraction_1(RI_1) + Packing\ Fraction_2(RI_2)$$

$$RI_{face\ down} = 0.39(2.3) + 0.61(1.45) = 1.78$$

$$RI_{edge\ up} = 0.49(2.3) + 0.51(1.45) = 1.87$$

The estimated RI was used in modeling the angular emission signal for a given transition dipole moment angle.

UV-vis spectrophotometry

Absorbance and relative photoluminescence spectra were collected using a Stellarnet BLACK-Comet UV-vis reconfigurable spectrometer. For colloidal absorbance and PL measurements, a broad-spectrum Stellarnet SL-5 Halogen/Deuterium light source is used for absorbance measurements, and a 392 nm LED light source 90° from the detector is used for photoluminescence measurements. A StarnaCells quartz cuvette with a path length of 1 cm was used. For film PL spectra, a 405 nm CW laser (30mA, 2 mW) was used to excite thin film samples mounted on a glass slide and a Thorlabs FEL0450 450 nm longpass filter was used as the laser was mounted normal to the plane of detection.

Sublayer screening

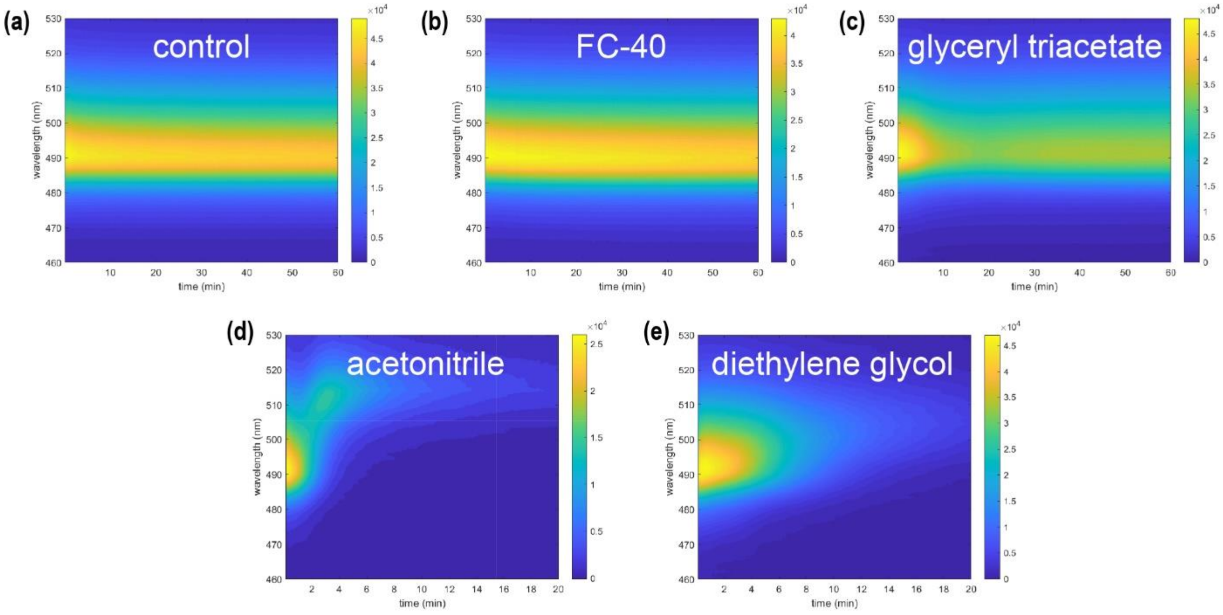

Figure S2. (a) Control sample: photoluminescence spectra over time for $CsPbBr_3$ nanoplates in hexane under constant stirring in air. Photoluminescence spectra over time for $CsPbBr_3$ nanoplates in hexane under constant stirring after the addition of immiscible sublayer candidate (1% by volume): (b) perofluorodecalin (FC-40), (c) glyceryl triacetate (GTA), (d) acetonitrile (ACN), and (e) diethylene glycol (DEG).

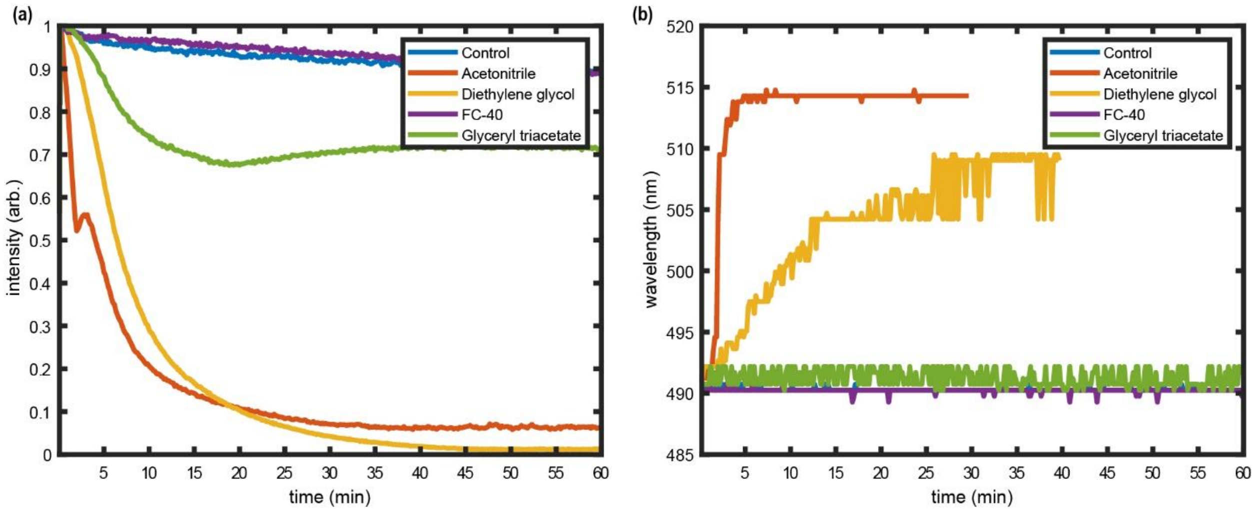

Figure S3. (a) Normalized maximum photoluminescence intensity and (b) center emission wavelength of $CsPbBr_3$ nanoplates in hexane after the addition of immiscible sublayer candidate (1% by volume). Redshifting of photoluminescence maximum is indicative of particle fusing, as seen in acetonitrile and diethylene glycol. Only perofluorodecalin and glyceryl triacetate maintain consistent center emission wavelength and >70% photoluminescence intensity.

Thin film preparation via assembly at the liquid-air interface

Assembly at the liquid-air interface was done in glass petri dishes (60 mm diameter, 15 mm height). Petri dishes and substrates were inspected for dust and cleaned before use. First, approximately 23 mL glyceryl triacetate (GTA) was added to the petri dish with the desired concentration of unbound ligand additive (0 to 0.68 mM OA and 0 to 0.66 mM OAm). We found that using higher concentrations of ligand resulted in macroscopic aggregation instead of a monolayer film. The surface of the sublayer was inspected for dust and stamped using a sacrificial cover slip to remove any microscopic contaminants on the liquid surface. Then, a suspension of $CsPbBr_3$ nanoplates in hexane (dilution standardized to 1.75 OD at 335 nm, around 100x diluted for a typical synthesis) was deposited onto the surface of the GTA to form two phases and was quickly covered with the top of the petri dish until all hexane had completely evaporated (less than 5 minutes).

The assembled film was transferred to the desired substrate by stamping the substrate onto the surface of the GTA. The substrates used in this study were either TEM grides or untreated 22x22 mm borosilicate glass coverslips. This was done using reverse action tweezers to hold TEM grids or wide mouth tweezers or a cover slip vacuum tool to hold cover slips. Once the films had been transferred to the substrates, substrates were dried under vacuum to remove any excess GTA that remained on the surface.

While this method produced large scale, highly oriented films of perovskite nanoplates, it was extremely sensitive to outside forces. For the effects of interfacial energy and vdW forces to control assembly regime, any other forces on the system had to be painstakingly minimized. Of note, movement of the surrounding gas of the environment had to be minimized as this was repeatedly observed to disrupt ordering of the particles. Similarly, any flow or bulk movement of the GTA or dust particles on the surface disrupted ordered assembly thoroughly. Thus, preparation was done inside an Ar glovebox, and glovebox filters were momentarily turned off during deposition of the nanoparticle suspension and substrate stamping to minimize movement of gas in the environment. Additionally, GTA was allowed to

sit undisturbed for at least 1 minute before depositing the nanoparticles to minimize movement in the sublayer.

The GTA itself also caused some degradation of the nanoplates as revealed by sublayer screening study. Evident in Figure 2 (c), as a result, quasi-spherical aggregates are shown interspersed with the nanoparticle ensemble causing nanoscale interruptions to the film. After assembly on the liquid sublayer, the delicate assembly remained sensitive to aggregation and tearing when transferring to a solid substrate. Excess GTA droplets on the surface caused a coffee-ringing effect upon drying. The film could be easily torn while manually transferring it onto the substrate, and in particular, the hydrophilic nature of borosilicate glass causes repulsion of the non-polar capping ligands thereby causing disruptions to the nanocrystal assemblies. Surface treatments of the glass substrate may minimize this and enable even larger assemblies.

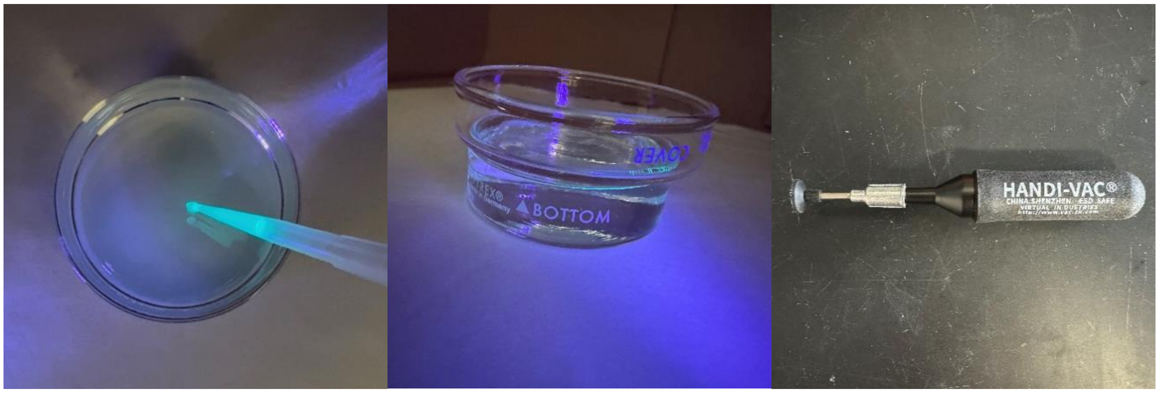

Figure S4. Photos showing (a) the addition of nanoplate suspension onto GTA sublayer, (b) covered petri dish during evaporation of native solvent, and (c) coverslip vacuum tool used to aid in the even stamping of glass onto the liquid sublayer.

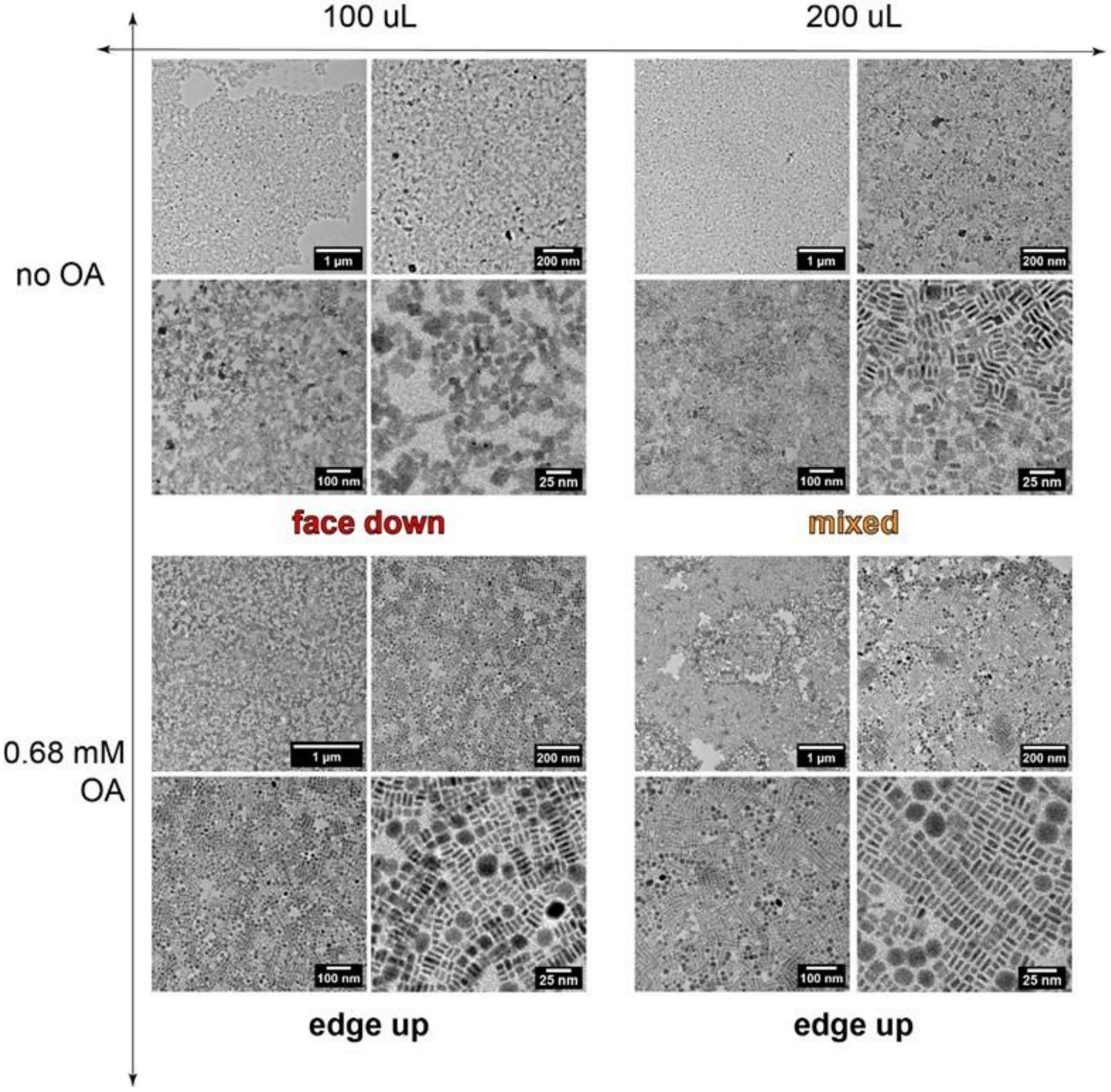

Figure S5. Transmission electron micrographs showing a summary of monolayer assembly regimes that can be achieved by tuning volume fraction and oleic acid addition. Images in the lower right quadrant for each condition are digitally cropped from images in the lower left quadrant for each condition.

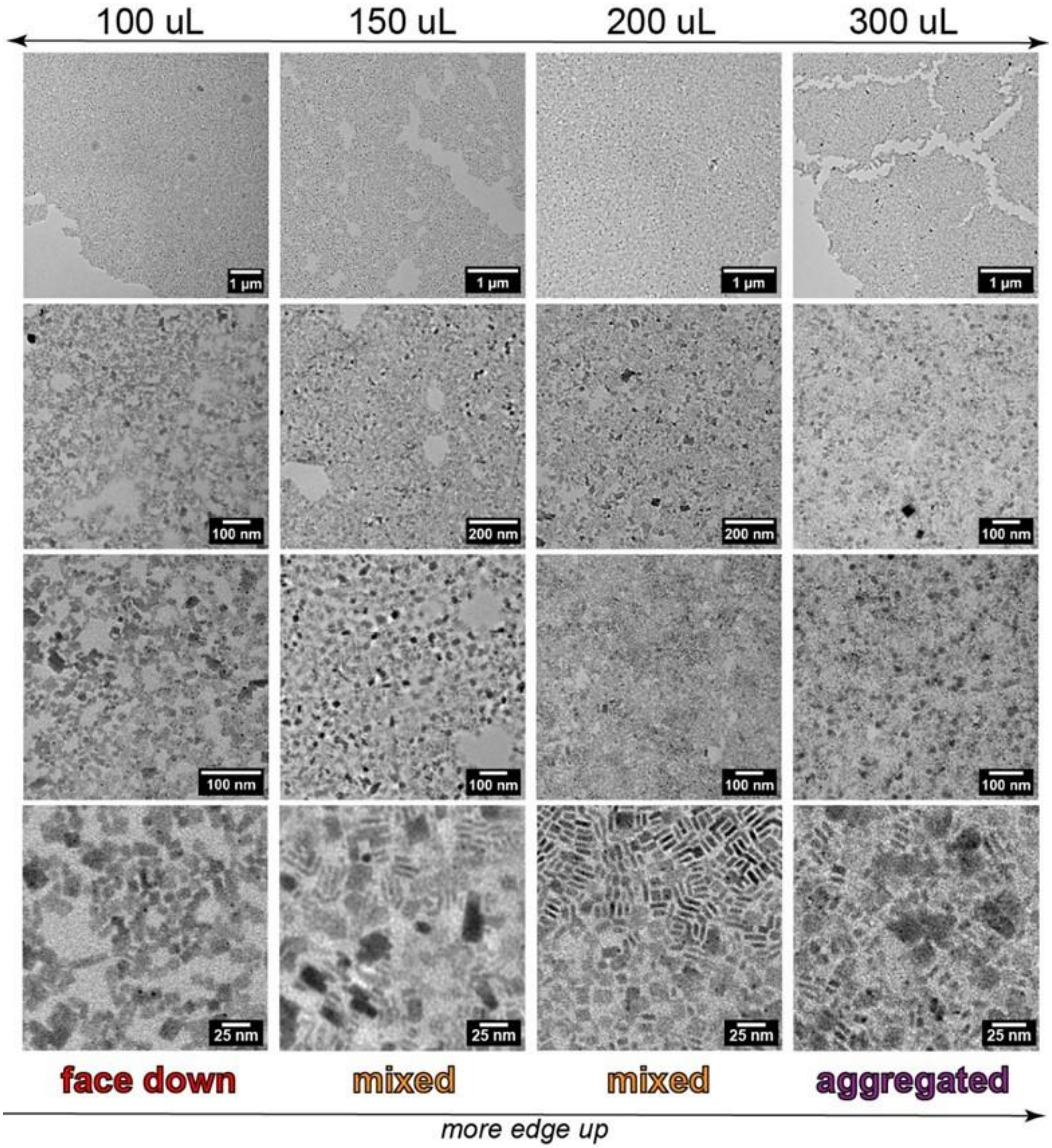

Figure S6. Transmission electron micrographs showing the evolution of assembly regime as the volume of nanoparticle suspension—and therefore total volume fraction of nanoparticles in the assembly—is increased from 100 to 300 μL. Images in row 4 are digitally cropped from images in row 3. In general, higher volume fraction yielded more edge up particles.

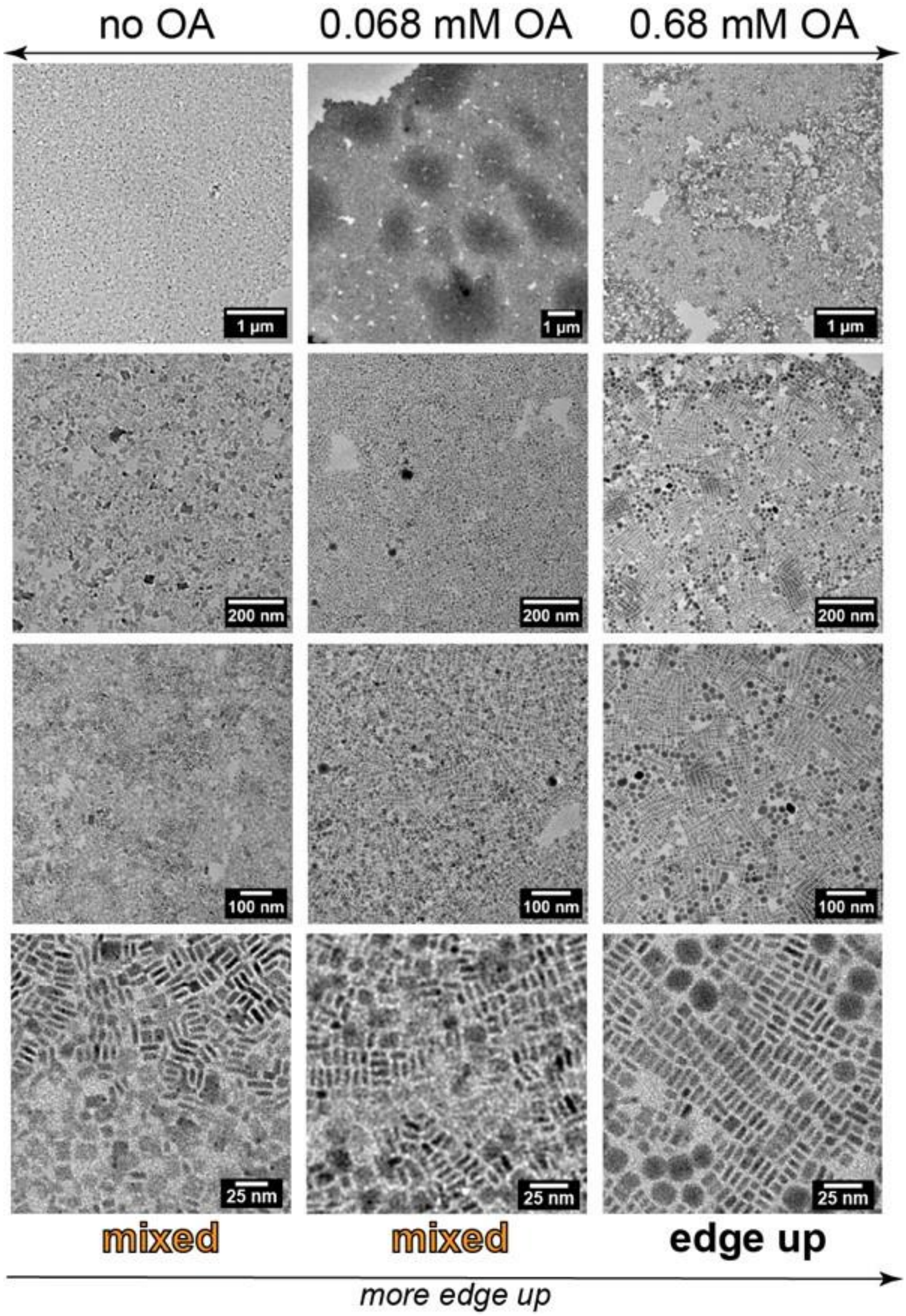

Figure S7. Transmission electron micrographs showing the evolution of assembly regime as the concentration of oleic acid in the sublayer is increased from 0 to 0.68 mM OA using 200 μL nanoplate suspension. Images in row 4 are digitally cropped from images in row 3. In general, more OA ligand added to the sublayer yielded more edge up particles.

Previous work by Gao et al.[7] reported ligand-directed assembly of only OA-capped CdSe nanoplates. We hypothesized that tuning the vdW interactions between both capping ligands in our system, oleic acid and oleylamine (OAm), might give finer control over the regime of assembly. TEM characterization of assemblies directed by OA + OAm addition are shown as Figure S8. We observed that adding both OA and OAm to the sublayer (0.68 mM OA and 0.66 mM OAm) yielded more undesirable

aggregation than OA alone. Assemblies showed overlapping and multiple layers of plates and film bunching. Reducing the concentration of the unbound ligand concentration tenfold (0.068 mM OA and 0.066 mM OAm) yielded a monolayer film but the orientation was still clearly within the mixed regime, as evidenced by the presence of both face down and edge up nanoplates. This was likely due to the amine group (NH_2) of the OAm and the carboxylic acid (COOH) of the OA having opposite effects on the interfacial energy, or the reaction of these two species in solution.

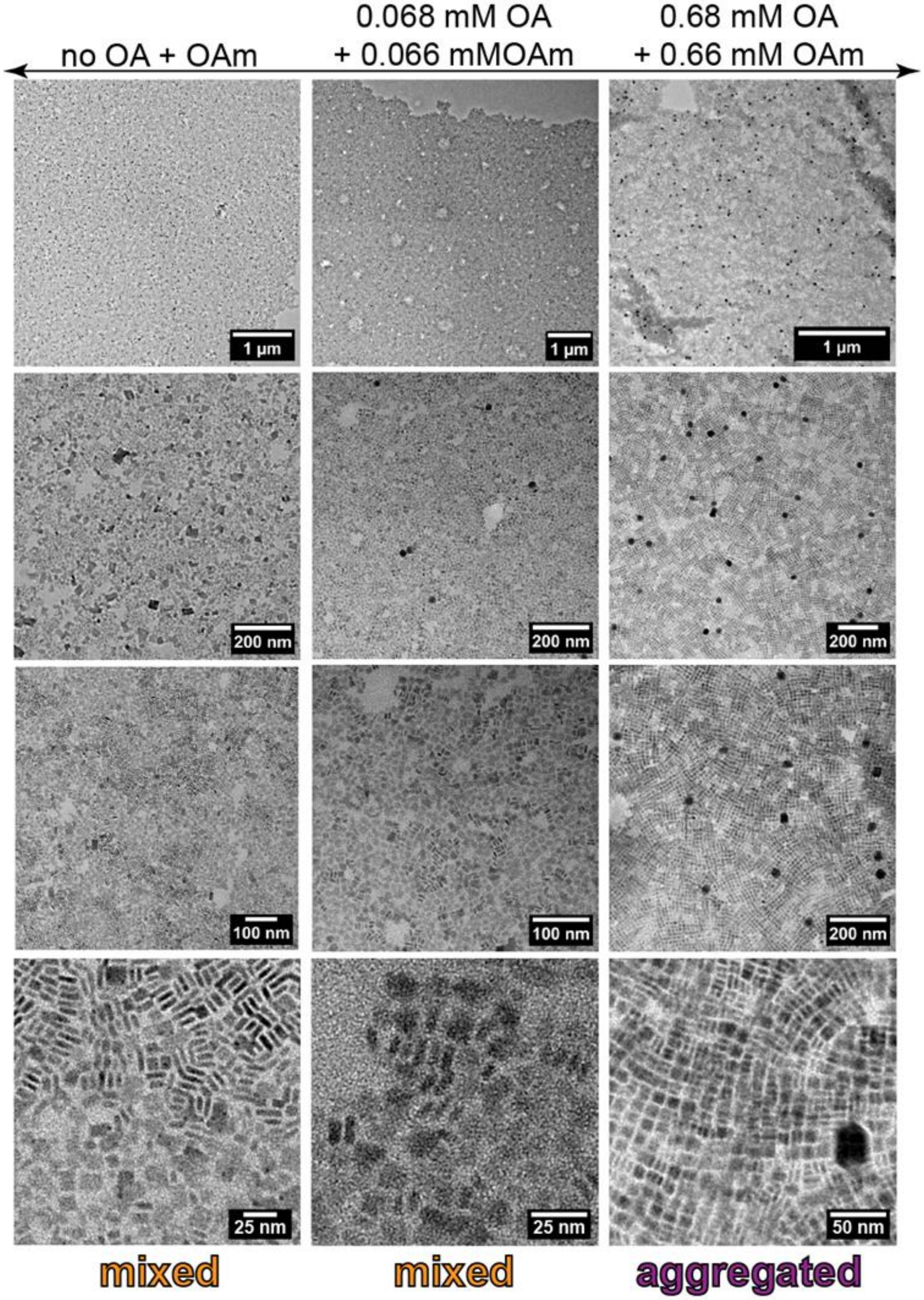

Figure S8. Transmission electron micrographs showing the evolution of assembly regime as the concentration of oleic acid plus oleylamine in the sublayer is increased from 0 to 0.68 mM OA + 0.66 mM OAm using 200 μL nanoplate suspension. Images in row 4 are digitally cropped from images in row 3.

Atomic Force Microscopy

Measurements were collected using a Bruker Dimension FastScan Atomic Force Microscope in ScanAsyst mode. A ScanAsyst-Air-HPI probe was used.

To determine the thickness of the nanoplate assemblies, the image was plane-leveled, row-aligned, and masked via height threshold to isolate the islands of nanocrystals from the background. Masks are shown in Figure S9 for face down samples and Figure S10 for edge up samples. The average height of the masked region was subtracted from the average height of the background.

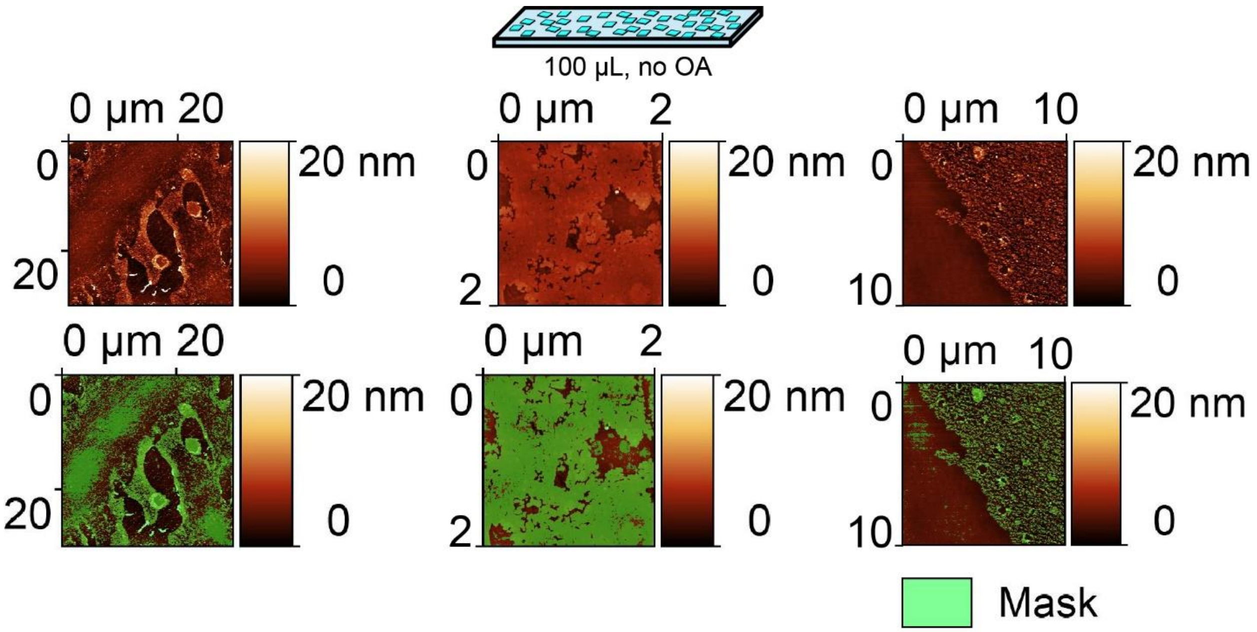

Figure S9. AFM showing face down assemblies, from which an average height of 3.4 nm was extracted using masked regions (green) and subtracted background.

.

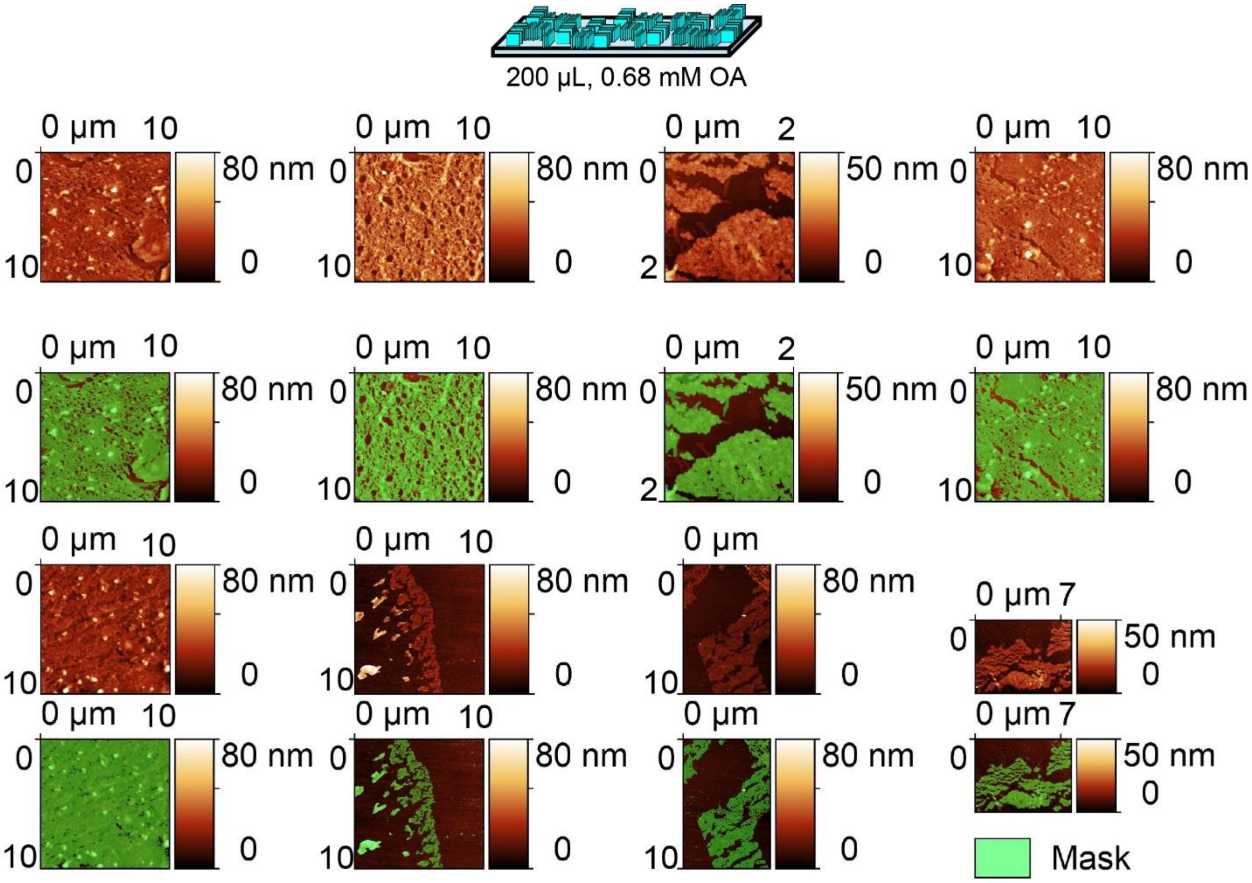

Figure S10. AFM showing edge up assemblies, from which an average height of 15.2 nm was extracted using masked regions (green) and subtracted background.

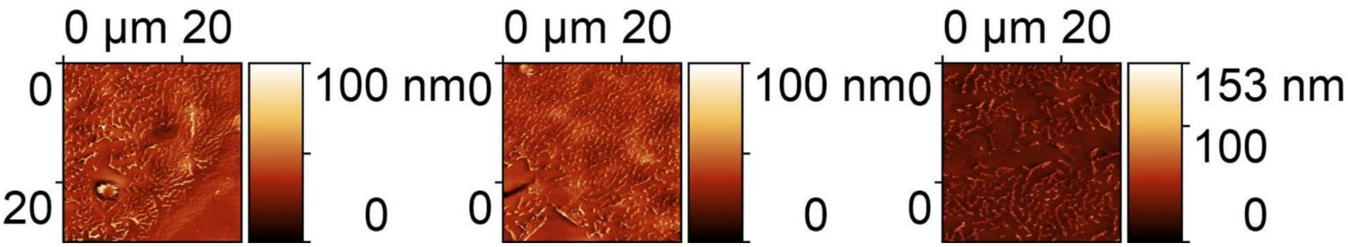

Figure S11. AFM showing micron-scale aggregation commonly observed in edge up samples and on glass substrates.

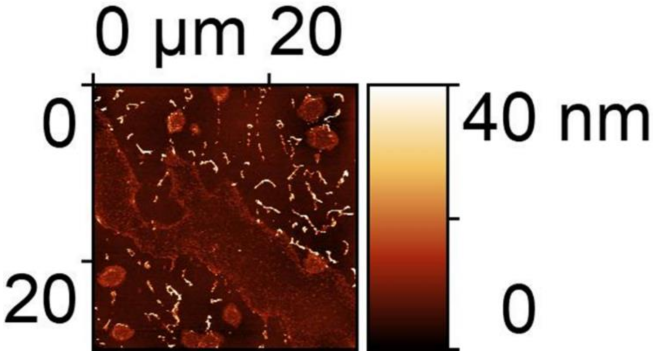

Figure S12. AFM showing coffee ringing-type aggregation that occurs from excess GTA on substrate.

Assembly extended to $CsPbBr_3$ nanocubes with DDAB ligand

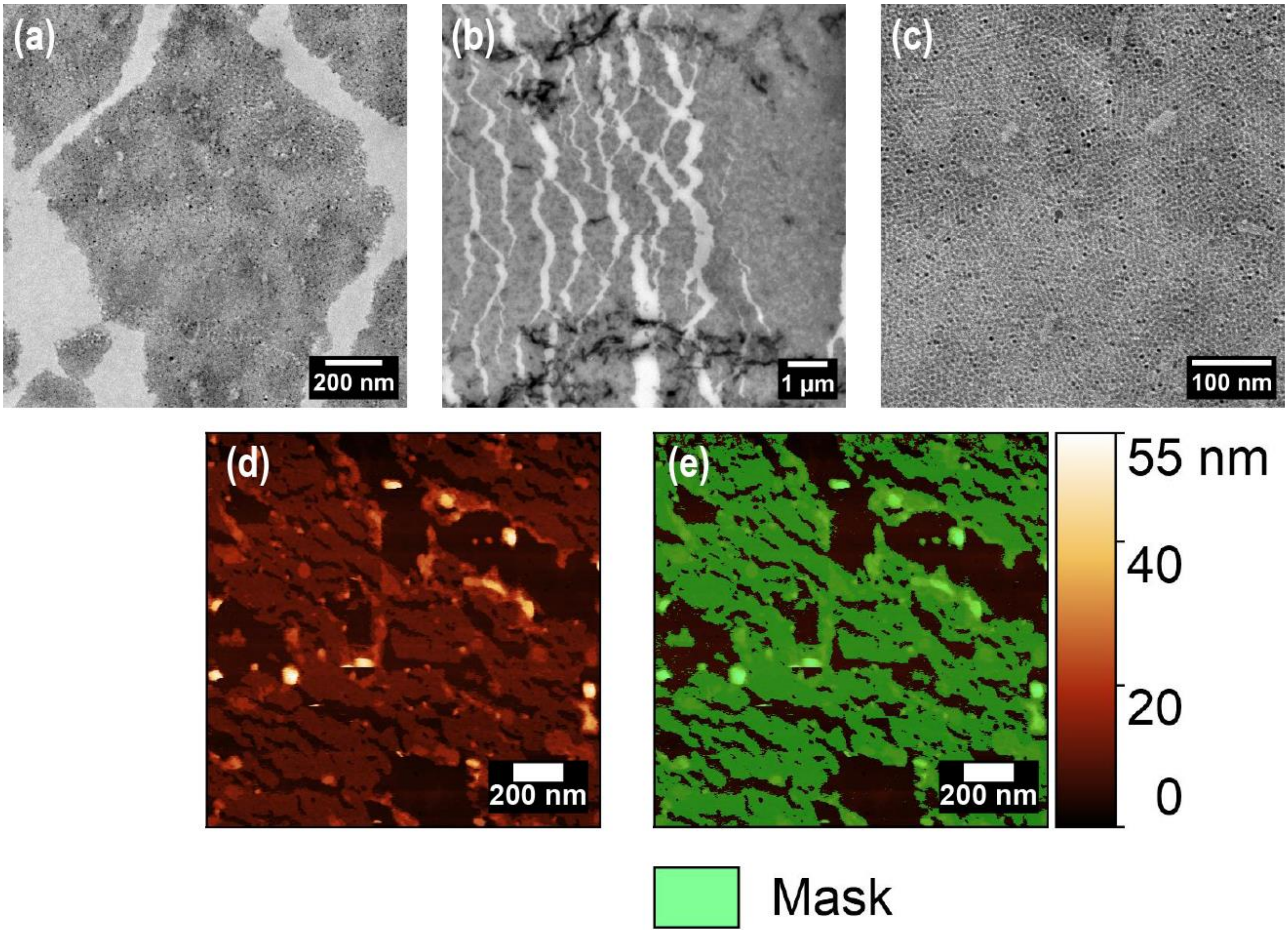

Figure S13. (a)-(c) TEM showing DDAB-stabilized $CsPbBr_3$ nanocubes assembled at the liquid-air interface diluted 40x from reaction concentration in heptane.No lignand was added to the subphase. (d)-(e) AFM showing nanocube assemblies from which an average height of 6.89 nm was extracted (nanocube diameter is 7.4 nm from TEM).[8]

Back Focal Plane (BFP) Fluorescence Microscopy

Emission from thin film samples on a glass coverslip was measured through the coverslip to determine the angular emission pattern at the air-glass interface.

A home-built inverted fluorescence microscope was used, including a Back Focal Plane imaging mode according to the procedure described in Kurvits et al. [9]The sample was excited by a ThorLabs LP405-SF10 405 nm laser diode (24 mA, 0.8 mW). This laser diode was collimated using a ThorLabs F810FC-405 collimation package and then expanded using a ThorLabs GBE02-A beam expander. A Semrock Di02-R405-25x36 25.2mm x 35.6 mm dichroic beamsplitter was used to direct the laser to the objective and minimize the transmission of any reflected laser light off the sample to the detector camera. The sample was excited through a UPlanSApo Olympus 100x oil-immersion objective (NA=1.45, Immersion Oil type F).

Fluorescence from the sample was collected by the objective and then passed through a tube lens (Thorlabs TTL180-A, f=180mm) to focus the signal onto a CCD camera for collection (Andor iXon Ultra 888 EMCCD). An additional filter (Chroma AT435LP 435 nm longpass filter) was placed before the camera to help cut out any remaining laser signal.

To transform the setup from fluorescence imaging into back focal plane imaging, a Bertrand lens (Thorlabs AC254-400-A-ML achromatic doublet) was placed between the beam splitter and tube lens as this changes the focal plane from the real-space sample to the back focal plane within the objective, creating a projection of the 3D angular emission pattern on the camera. [9] A linear polarizer (Thorlabs LPVISE100-A) was also added after the beamsplitter to polarize the back focal plane image and simplify the extraction of the transition dipole moment angle. All BFP images were generated using 1s exposure times. 5 images were collected at ≥7 sample areas.

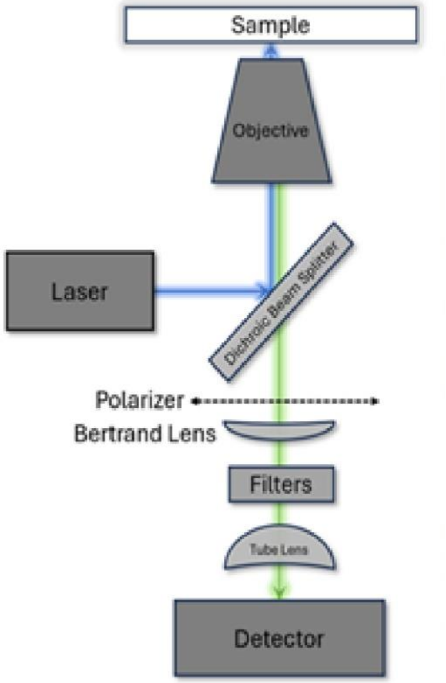

Figure S14. Schematic of back focal plane microscope. Reproduced with permission from Russ et al. [10] Copyright 2025 *ACS Photonics*.

Modeling angle-resolved light emission

The dipole orientation factor (Θ) is the relative dipole strength (*p*) of in-plane or parallel dipoles to all dipoles: [11]

$$\Theta = \frac{p_{\parallel}}{p_{\parallel}+p_{\perp}} \tag{S1}$$

The orientation factor and effective transition dipole moment (TDM) angle (relative to substrate) are related by[12]

$$TDM = arccos(\sqrt{\Theta}) \tag{S2}$$

The angular light emission pattern from these dipoles within a thin film can be described by a three layer model where the top layer (n_1) is air, the middle layer (n_2) is the emissive film, and the bottom layer (n_3) is the glass substrate.[7,13–15] The horizontal dipole contribution along x is eliminated by linear polarizer in experiment, which allows us to decouple horizontal and vertical dipole contributions when fitting to experimental data. Polarized light emission intensity projected onto a 2-D collection plane (N_{pol}) as a function of photon momentum vectors along the x- and y-directions (k_x, k_y) is defined as:[15,16]

$$N^{pol}(k_x, k_y) = C\left[\frac{1}{2}\left(\rho_{IP}^{p} + \rho_{IP}^{s}\right)\cos^2(TDM) \;\; + \rho_{OP}^{p}\sin^2(TDM)\;\right] \tag{S3}$$

or, in terms of orientation factor:

$$N^{pol}(k_x, k_y) = C\left[\frac{1}{2}\left(\rho_{IP}^{p} + \rho_{IP}^{s}\right)\Theta \;\; + \rho_{OP}^{p}(1-\Theta)\;\right] \tag{S4}$$

where C is a normalization constant. ρ_{ip}^{s}, ρ_{ip}^{p}, ρ_{op}^{p} are photonic density of states in the *s*- and *p*-polarized, in-plane and out-of-plane directions governed by the thickness, refractive index, and emission wavelength of the emissive layer. Density of states in each direction are given by:

$$\rho_{IP}^{s} = \rho_{y}^{s}(k_x, k_y) = \left(\frac{1}{8\pi k_0^2}\right)\left(\frac{k_0}{k_{z,3}}\right)\left|\frac{t_{32}^{s}e^{\frac{ik_{z,2}D}{2}}\left(1+r_{21}^{s}e^{ik_{z,2}D}\right)}{1-r_{21}^{s}r_{23}^{s}e^{2ik_{z,2}D}}\frac{k_y}{\sqrt{k_x^2+k_y^2}}\right|^2 \tag{S5}$$

$$\rho_{IP}^{p} = \rho_{x}^{p}(k_x, k_y) = \left(\frac{1}{8\pi k_0^2}\right)\left(\frac{k_0}{k_{z,3}}\right)\left|\frac{t_{32}^{p}e^{\frac{ik_{z,2}D}{2}}\left(1-r_{21}^{p}e^{ik_{z,2}D}\right)\frac{k_{z,2}}{n_2k_0}}{1-r_{21}^{p}r_{23}^{p}e^{2ik_{z,2}D}}\frac{k_x}{\sqrt{k_x^2+k_y^2}}\right|^2 \tag{S6}$$

$$\rho_{OP}^{p} = \rho_{z}^{s}(k_x, k_y) = \left(\frac{1}{8\pi k_0^2}\right)\left(\frac{k_0}{k_{z,3}}\right)\left|\frac{t_{32}^{p}e^{\frac{ik_{z,2}D}{2}}\left(1+r_{21}^{p}e^{ik_{z,2}D}\right)\frac{k_x}{n_2k_0}}{1-r_{21}^{p}r_{23}^{p}e^{2ik_{z,2}D}}\right|^2 \tag{S7}$$

where

$$k_{z,i} = \sqrt{n_i^2k_0^2 - \left(k_x^2 + k_y^2\right)} \tag{S8}$$

$$k_0 = \frac{2\pi}{\lambda} \tag{S9}$$

λ is the wavelength of the emitted light, n_i is the refractive index of layer i, t_{ij}^{l} and r_{ij}^{l} are the Fresnel transmission and reflection coefficients between layers i and j for l-polarized light, and D is the thickness of the emissive layer. The Fresnel coefficients for s and p polarized light are given by[7,14]

$$t_{ij}^{p} = \frac{2n_i n_j k_{zi}}{n_j^2 k_{zi} + n_i^2 k_{zj}};\ t_{ij}^{s} = \frac{2k_{zi}}{k_{zi} + k_{zj}};\ r_{ij}^{p} = \frac{n_j^2 k_{zi} - n_i^2 k_{zj}}{n_j^2 k_{zi} + n_i^2 k_{zj}};\ r_{ij}^{p} = \frac{k_{zi} - k_{zj}}{k_{zi} + k_{zj}} \tag{S10}$$

We note that light emission in polar coordinates ($N^{pol}(\theta, \phi)$), where θ is the polar angle and ϕ is the azimuthal angle, is related to light emission in k-space by

$$k_x = k_0 n_3\ sin\, sin\ (\phi)\ \ sin\, sin\ (\theta)\ ;\ k_y = k_0 n_3\ sin\, sin\ (\phi)\ \ cos\, cos\ (\theta) \tag{S11}$$

This model for emission signal is compared to our measured BFPFM data, and the effective TDM of best fit is determined by $\chi 2$ minimization according to the procedure described in Russ et al.[10] Table S1 gives the physical parameters used to model each sample condition when fitting TDM alignment where 0 nm is an accepted approximation for film thicknesses much smaller than the wavelength of light in which Fresnel reflections are modeled but phase shift is neglected.[4,7]

Table S1. Properties of emitting layer describing the BFP signal for each sample condition

	λ (nm) (film PL)	RI (TEM using EMA)	D (nm) (AFM)	D (nm) (TEM)	D (nm) (model input)
100 µL, no OA	492	1.78	3.4	4 ±1	0
200 µL, 0.68 mM OA	492	1.87	15.2	14±3	15

Background signal noise across datasets had an estimated standard deviation of 2.31% which results in an associated inherent uncertainty of fit. According to the confidence intervals reported by Russ et al. [10], the inherent uncertainty in the fit of our measurements given the thickness, RI of the emitting layer, and standard deviation of background noise levels is less than ±0.1°.

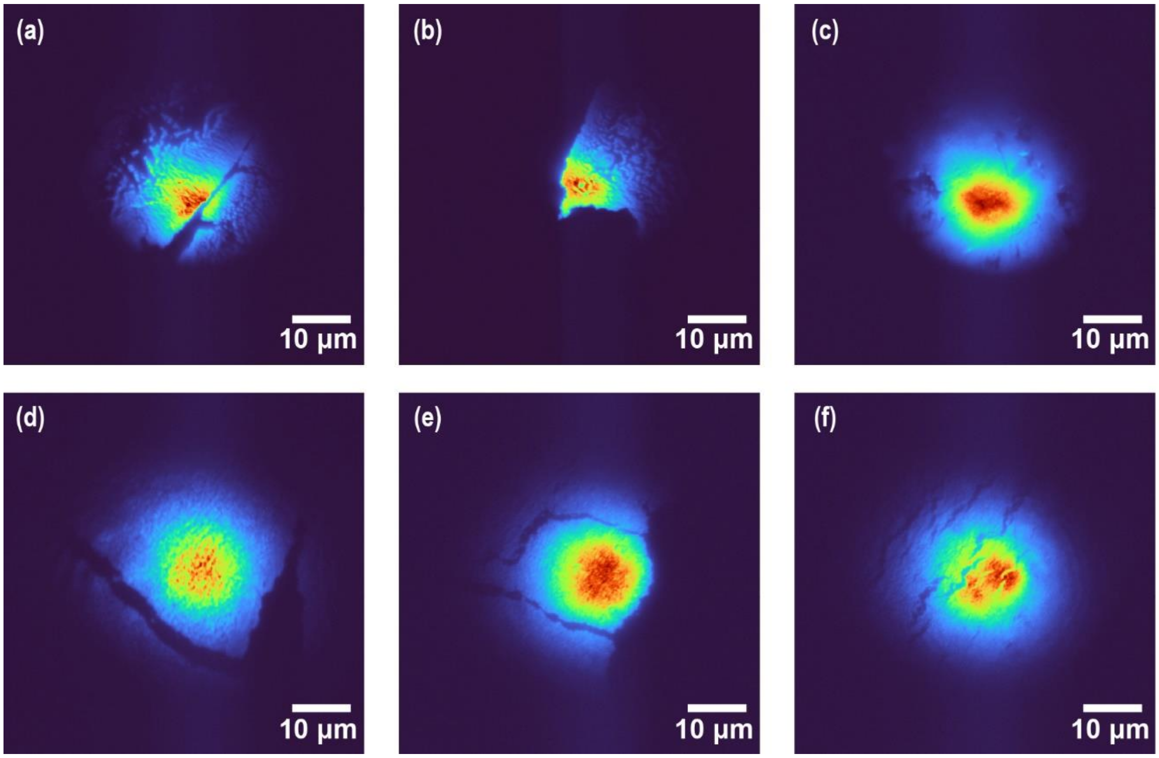

Figure S15. Fluroescence microscopy images of (a)-(c) edge up assemblies and (d)-(f) face down assemblies with an approximate scalebar of 10 µm.

Poor fits of BFPFM data indicating thickness variability and defects in assemblies

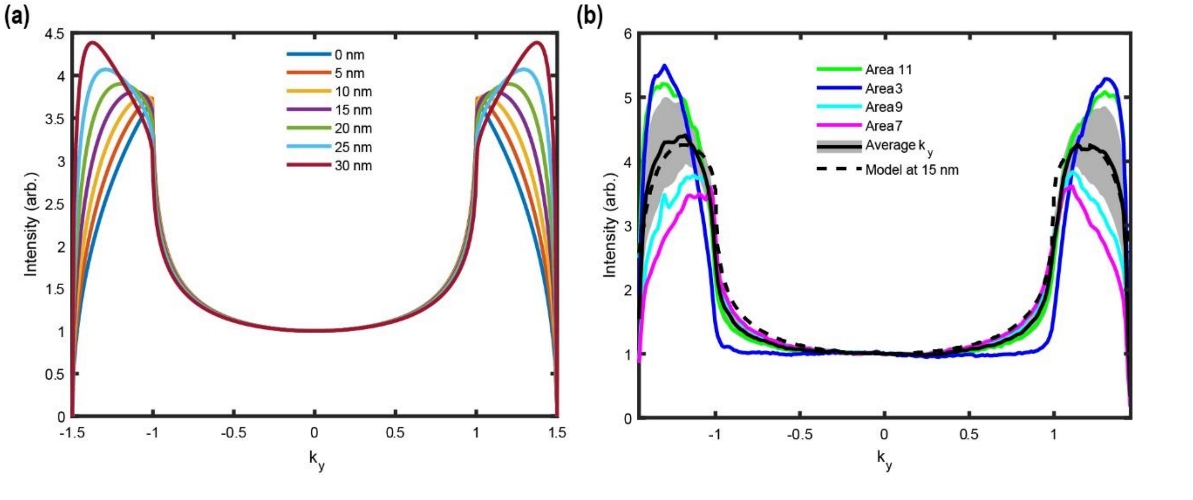

Figure S16. (a) Modeled k_y cross section where k_x=0 for varying thicknesses showing how the k-space location of $|k_{y,\text{max intensity}}|$ is pushed higher for increasing thicknesses. (b) k_y cross sections for collected data that showed a poor fit to 15 nm, indicating thickness variation edge up samples. The gray shading denotes one standard deviation from the average k_y across measured areas.

Effect of surface charging on nanoplate TDM

To further quantify the effects of surface charging versus the effects of confinement and orientation, we report observed TDM as compared to the theoretical TDM calculated from particle geometry (Figure S17 (a)). We also note that the percent by which the vertical TDM is exaggerated over the modeled value in a vacuum[14] is directly correlated with the glass contact area to particle volume ratio (Figure S17 (b)).

Table S2. Predicted TDM values in a vacuum

Geometry	Predicted TDM	Source
EU plate, this work	43.5°	calculated from Jurow et al.[14]
cube, Jurow et al. [14]	35.3°	equal dipole contributions
cube, Parsons et al.[8]	35.3°	equal dipole contributions
FD plate, this work	13°	Jurow et al.[14]
FD plate, Jurow et al.[14]	12°	Jurow et al.[14]

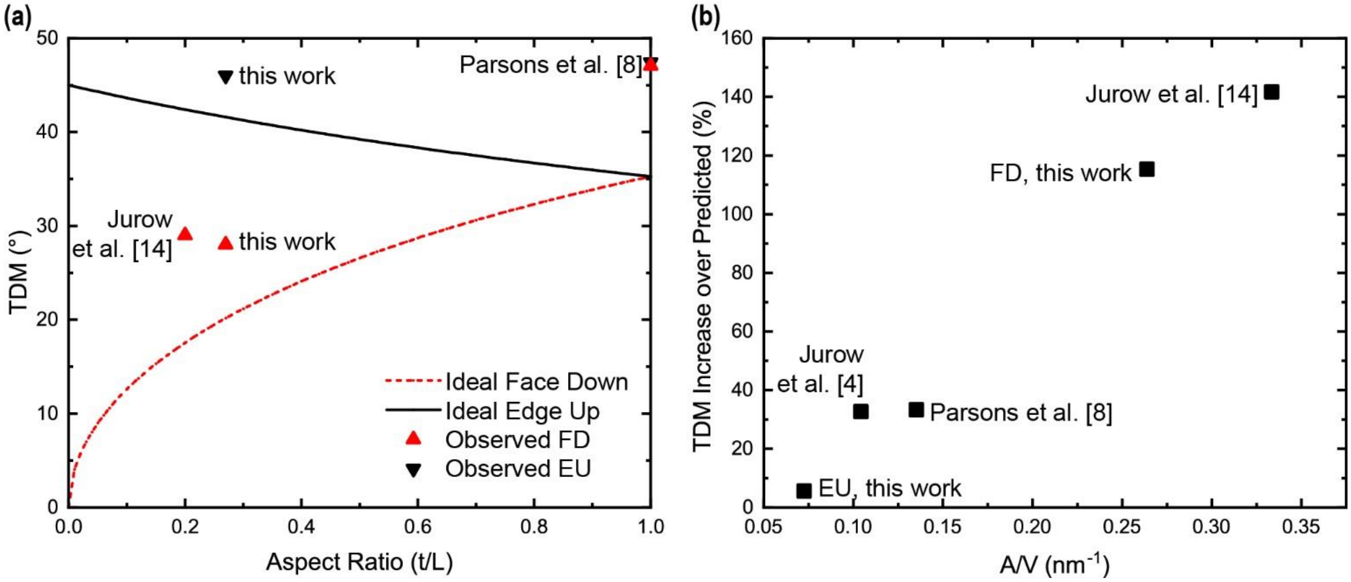

Figure S17. (a) Ideal theoretical (geometric, agnostic of surface response) and observed dipole contribution for 2D or quasi-2D $CsPbBr_3$ nanoplate films in the face down or edge up regimes. (b) % by which TDM on glass increases over the predicted TDM in a vacuum, modeled by DFT by Jurow et al.[14], versus ratio of particle contact area with glass (nm^2) to total particle volume (nm^3).

References

(1) Bertolotti, F.; Nedelcu, G.; Vivani, A.; Cervellino, A.; Masciocchi, N.; Guagliardi, A.; Kovalenko, M. V. Crystal Structure, Morphology, and Surface Termination of Cyan-Emissive, Six-Monolayers-Thick CsPbBr3 Nanoplatelets from X-Ray Total Scattering. *ACS Nano* **2019**, *13* (12), 14294–14307. https://doi.org/10.1021/acsnano.9b07626.

(2) Bekenstein, Y.; Koscher, B. A.; Eaton, S. W.; Yang, P.; Alivisatos, A. P. Highly Luminescent Colloidal Nanoplates of Perovskite Cesium Lead Halide and Their Oriented Assemblies. *J Am Chem Soc* **2015**, *137* (51), 16008–16011. https://doi.org/10.1021/jacs.5b11199.

(3) Brown, A. A. M.; Mathews, N.; Vashishtha, P.; Hooper, T. J. N.; Ng, Y. F.; Nutan, G. V.; Fang, Y.; Giovanni, D.; Tey, J. N.; Jiang, L.; Damodaran, B.; Sum, T. C.; Pu, S. H.; Mhaisalkar, S. G. Precise Control of CsPbBr3 Perovskite Nanocrystal Growth at Room Temperature: Size Tunability and Synthetic Insights. *Chemistry of Materials* **2021**, *33* (7), 2387–2397. https://doi.org/10.1021/acs.chemmater.0c04569.

(4) Jurow, M. J.; Lampe, T.; Penzo, E.; Kang, J.; Koc, M. A.; Zechel, T.; Nett, Z.; Brady, M.; Wang, L. W.; Alivisatos, A. P.; Cabrini, S.; Brütting, W.; Liu, Y. Tunable Anisotropic Photon Emission from Self-Organized CsPbBr3 Perovskite Nanocrystals. *Nano Lett* **2017**, *17* (7), 4534–4540. https://doi.org/10.1021/acs.nanolett.7b02147.

(5) Yakunin, S.; Protesescu, L.; Krieg, F.; Bodnarchuk, M. I.; Nedelcu, G.; Humer, M.; De Luca, G.; Fiebig, M.; Heiss, W.; Kovalenko, M. V. Low-Threshold Amplified Spontaneous Emission and Lasing from Colloidal Nanocrystals of Caesium Lead Halide Perovskites. *Nature Communications 2015 6:1* **2015**, *6* (1), 1–9. https://doi.org/10.1038/ncomms9056.

(6) Bernardo-Gil, G.; Esquivel, M.; Ribeiro, A. *Densities and Refractive Indices of Pure Organic Acids as a Function of Temperature*; 1990; Vol. 35. https://pubs.acs.org/sharingguidelines.

(7) Gao, Y.; Weidman, M. C.; Tisdale, W. A. CdSe Nanoplatelet Films with Controlled Orientation of Their Transition Dipole Moment. *Nano Lett* **2017**, *17* (6), 3837–3843. https://doi.org/10.1021/acs.nanolett.7b01237.

(8) Parsons, L.; Russ, B.; Eisler, C. Tunable Angular Light Emission of Lead Halide Perovskite Nanocrystal Thin Films via Solution-Processed Substrate Treatment. *ACS Nanoscience Au* **2025**. https://doi.org/10.1021/acsnanoscienceau.5c00054.

(9) Kurvits, J. A.; Jiang, M.; Zia, R. Comparative Analysis of Imaging Configurations and Objectives for Fourier Microscopy. *Journal of the Optical Society of America A* **2015**, *32* (11), 2082. https://doi.org/10.1364/JOSAA.32.002082.

(10) Russ, B.; Lin, T.-T.; Elenteny, H.; Eisler, C. N. Quantifying the Accuracy and Precision of the Transition Dipole Moment Alignment from Realistic Angular Emission Data. *ACS Photonics* **2025**. https://doi.org/10.1021/acsphotonics.5c00684.

(11) Kim, K.-H.; Kim, J.-J.; Kim, K.-H.; Kim, J.-J. Origin and Control of Orientation of Phosphorescent and TADF Dyes for High-Efficiency OLEDs. *Advanced Materials* **2018**, *30* (42), 1705600. https://doi.org/10.1002/ADMA.201705600.

(12) Hofmann, A.; Schmid, M.; Brütting, W. The Many Facets of Molecular Orientation in Organic Optoelectronics. *Adv Opt Mater* **2021**, *9* (21), 2101004. https://doi.org/10.1002/ADOM.202101004.

(13) Lieb, M. A.; Zavislan, J. M.; Novotny, L. Single-Molecule Orientations Determined by Direct Emission Pattern Imaging. *Journal of the Optical Society of America B* **2004**, *21* (6), 1210. https://doi.org/10.1364/JOSAB.21.001210.

(14) Jurow, M. J.; Morgenstern, T.; Eisler, C.; Kang, J.; Penzo, E.; Do, M.; Engelmayer, M.; Osowiecki, W. T.; Bekenstein, Y.; Tassone, C.; Wang, L. W.; Alivisatos, A. P.; Brütting, W.; Liu, Y. Manipulating the Transition Dipole Moment of CsPbBr3 Perovskite Nanocrystals for Superior Optical Properties. *Nano Lett* **2019**, *19* (4), 2489–2496. https://doi.org/10.1021/acs.nanolett.9b00122.

(15) Taminiau, T. H.; Karaveli, S.; Hulst, N. F. Van; Zia, R. Quantifying the Magnetic Nature of Light Emission. *Nat Commun* **2012**, *3*, 976–979. https://doi.org/10.1038/ncomms1984.

(16) Schuller, J. A.; Karaveli, S.; Schiros, T.; He, K.; Yang, S.; Kymissis, I.; Shan, J.; Zia, R. Orientation of Luminescent Excitons in Layered Nanomaterials. *Nat Nanotechnol* **2013**, *8* (4), 271–276. https://doi.org/10.1038/nnano.2013.20.
